# A Spatially Distributed Microneedle System for Bioorthogonal T Cell‐Guided Cancer Therapy

**DOI:** 10.1002/advs.202416841

**Published:** 2025-02-08

**Authors:** Lanya Li, Fei Wang, Shushan Mo, Junyao Deng, Xueyi Wang, Jiacong Ai, Yingxian Xiao, Yan Zeng, Qishan Li, Yixin Zhang, Limin Cai, Zhenhua Li

**Affiliations:** ^1^ The Tenth Affiliated Hospital, Southern Medical University (Dongguan People's Hospital) Dongguan 523059 China; ^2^ Dongguan Key Laboratory of Basic, Clinical and Digital Research on Common Orthopedic Diseases Dongguan 523059 China; ^3^ College of Pharmaceutical Science Key Laboratory of Pharmaceutical Quality Control of Hebei Province Hebei University Baoding 071002 China; ^4^ The First School of Clinical Medicine Southern Medical University Guangzhou 510515 China; ^5^ Guangdong Provincial Key Laboratory of Cardiac Function and Microcirculation Guangzhou 510515 China

**Keywords:** bioorthogonal reaction, lymph node, solid tumors, spatially distributed microneedle, T cell migration

## Abstract

Chimeric antigen receptor (CAR)‐T cell therapy represents a promising strategy for cancer treatment. However, the diversity of solid tumor antigens and the poor infiltration of CAR‐T cells significantly hinder the efficacy of CAR‐T therapies against tumors. Here, a spatially distributed microneedle system (SDMNS) is developed that leverages bioorthogonal reactions to activate and guide endogenous T cells to tumors for effective destruction. The SDMNS consists of two dissolving microneedles, each loaded with complementary bioorthogonal groups and applied separately to lymph nodes and tumor sites. One microneedle loaded with two dibenzocyclooctyne (DBCO)‐modified antibodies activates T cells and labels them with bioorthogonal groups in lymph nodes. The other microneedle, containing N‐azidoacetylmannosamine‐tetraacylated (Ac4ManNAz) for glycometabolic labeling of tumor cells, and the T cell chemotactic factor IP10, is applied directly to the tumor site. The in vivo studies demonstrate that SDMNS effectively directs the migration and infiltration of endogenous activated T cells into the tumors. Through a bioorthogonal click reaction, DBCO‐modified T cells conjugate with azide (N_3_)‐modified tumor cells, eliciting robust antitumor immune responses and durable immune memory. The SDMNS offers a novel strategy to overcomes tumor heterogeneity by facilitating the directed migration of endogenous T cells.

## Graphical abstract

A spatially distributed microneedle system (SDMNS) comprises two dissolving microneedles: DBCO‐αCD3/28@MNs, which activate T cells and label them with bioorthogonal groups in lymph nodes, and IP10‐Az@MNs, which label tumor cells and guide T cell migration. This innovative system utilizes bioorthogonal reactions to promote the interaction between activated endogenous T cells and tumor cells, facilitating targeted tumor killing.

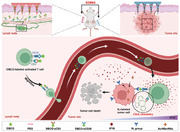

## 1 Introduction

CAR‐T cell therapy is a highly powerful and promising approach for cancer, particularly for CD19‐positive hematological malignancies.^[^ ^1^, ^2^, ^3^ ^]^ On this basis, numerous clinical trials have been undertaken to evaluate the effectiveness of CAR‐T cell therapy for solid tumors, though limited clinical benefit has been achieved so far.^[^ ^4^, ^5^, ^6^ ^]^ The effectiveness of T cell therapy for solid tumors relies on the stimulation of tumor‐specific T lymphocytes and their successful infiltration into the tumor tissue.^[^ ^7^ ^]^ However, inter‐ and intratumor cell antigen heterogeneity presents significant challenges to CAR efficacy in solid tumor, due to CAR‐T cells targeting specific antigens.^[^ ^8^, ^9^ ^]^ Even though CAR‐T cells are effective against tumor, the loss of target antigens can result in the development of tumor antigen escape variants.^[^ ^10^, ^11^ ^]^ Furthermore, the complex manufacturing process of CAR‐T cells, which involves autologous T‐cell isolation, CAR design, genetic modification with CAR, and ex vivo stimulation and expansion, limits their widespread application.^[^ ^12^ ^]^ The fundamental prerequisite for CAR‐T therapy is effective trafficking from blood into the tumor site, which is the first step to realize effector functions.^[^ ^13^, ^14^, ^15^, ^16^ ^]^ In vivo, CAR‐T cells must navigate high‐speed blood flow and extravasate from the vasculature to reach tumor tissues after intravenous infusion.^[^ ^17^, ^18^ ^]^ The dense extracellular matrix in solid tumors further hinders CAR‐T cell accumulation at the tumor site.^[^ ^19^ ^]^ Moreover, inefficient chemokine secretion within the tumor microenvironment hinders CAR‐T cell recruitment.^[^ ^20^, ^21^ ^]^ Even upon reaching the tumor site, CAR‐T cells cannot effectively recognize cancer cells and perform their effector functions due to the immunosuppressive tumor microenvironment.^[^ ^22^ ^]^ Therefore, it is essential to develop novel strategies to improve T cell targeting, trafficking, and infiltration into solid tumor tissues.

To date, most efforts to improve the tumor recognition efficacy of T cell therapy have centered on identifying more specific antigens.^[^ ^23^ ^]^ However, the search for novel tumor‐specific antigens is challenging and complicated due to a large degree of antigen heterogeneity. Leveraging chemical approaches to mediate interactions between tumor cells and T cells offers an alternative strategy to enhance T cell targeting and infiltration. Bioorthogonal reaction is a valuable tool for specifically labeling living cells and regulating cell–cell interactions under physiological conditions.^[^ ^24^, ^25^, ^26^, ^27^ ^]^ We have previously developed a pretargeting and bioorthogonal chemistry system to direct endogenous stem cells to cardiac injury sites for repair using two bioorthogonal antibodies administered intravenously with a 48 h interval.^[^ ^28^ ^]^ Recently, metabolic glycoengineering combined with bioorthogonal chemistry has demonstrated significant potential in dynamically controlling cell–cell interactions through a two‐step strategy.^[^ ^29^, ^30^, ^31^, ^32^ ^]^ Briefly, bioorthogonal groups (e.g., N_3_ groups) are incorporated into cellular glycans and displayed on cell surface via the biosynthesis of unnatural sugars. These bioorthogonal groups subsequently form stable and robust chemical bonds with the complementary group (e.g., DBCO) through strain‐promoted azide–alkyne cycloaddition (SPAAC) reaction. This method allows the efficient conjugation of probes, antibodies, or even cells with a DBCO group to azide‐functionalized cell surfaces. Moreover, these bioorthogonal reactions occur rapidly within organisms without interfering with native biochemical processes. Cai et al. applied this strategy to incorporate the bioorthogonal groups (*─*N_3_ and *─*BCN) onto the surface of CAR‐T cells and B‐lymphoma cells by glycometabolism of Ac4GalNAz and Ac4ManNBCN, respectively. The azide‐labeled CAR‐T cells exhibited enhanced infiltration, recognition, and selective cytotoxicity against BCN‐labeled B‐lymphoma cells through bioorthogonal chemistry‐mediated cell–cell interactions.^[^ ^33^ ^]^ Building on this approach, they further labeled NK cells and B‐lymphoma cells to augmented NK cells‐mediated tumor immunotherapy.^[^ ^34^ ^]^ Despite these advancements, several challenges remain. For example, these strategies rely on the ex vivo expansion and labeling of immune cells, followed by their reinfusion into the host. Additionally, most of these methods are specifically tailored to hematological malignancies, limiting their broader applicability.

**Figure 1:**
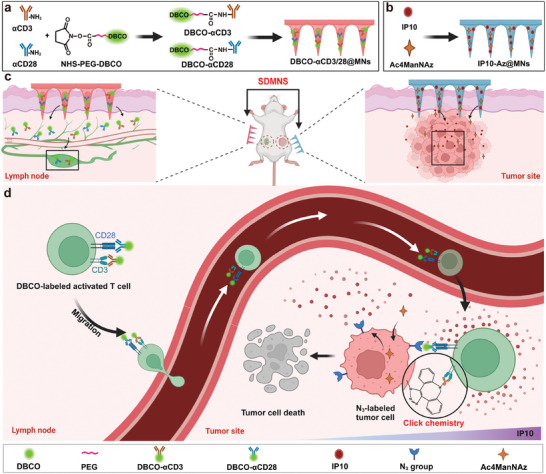
Schematic illustration of the SDMNS and its potential mechanism of antitumor immune responses. a) Fabrication of DBCO‐αCD3/28@MNs. αCD3 and αCD28 antibodies are modified with NHS‐PEG5000‐DBCO and encapsulated within a hyaluronic acid microneedle patch. b) Fabrication of IP10‐Az@MNs. Ac4ManNAz and IP10 are encapsulated within a hyaluronic acid microneedle patch. c) The SDMNS utilizes two distinct microneedles to label different cell types at separate sites. DBCO‐αCD3/28@MNs are applied to the lymph node region to activate and label T cells, while IP10‐Az@MNs is applied directly to the tumor site to label tumor cells. d) Simplified mechanism of SDMNS. Bioorthogonal reaction‐mediated T cell targeting enhances cancer immunotherapy by directing activated T cells to tumors for selective cytotoxicity.

Herein, we designed a spatially distributed microneedle system (SDMNS) based on bioorthogonal reaction to enable endogenous, activated T cell‐mediated antitumor immunity without the need for genetically modified T cells with tumor antigen‐specific T cell receptors (TCRs) or CARs (**Figure** **1**). Microneedles (MNs) represent a novel transdermal drug delivery system characterized by minimal invasiveness and painlessness.^[^ ^35^, ^36^, ^37^ ^]^ They have been extensively applied for local and systemic delivery of various agents, including small molecules, DNA, peptides, and proteins.^[^ ^38^, ^39^, ^40^ ^]^ MNs consist of microscale needle arrays capable of penetrating the stratum corneum to deliver drugs to the epidermis or dermis, which are rich in lymphatic vessels. Previous studies have demonstrated that this transdermal delivery system facilitates the trafficking of therapeutic cargo to lymph nodes via lymphatic vessels.^[^ ^41^, ^42^, ^43^ ^]^ Lymph nodes are critical to the success of immune therapies, as they serve as primary sites for immune cell activation. To activate T cells and label them with bioorthogonal groups in situ, we developed DBCO‐αCD3/28@MNs, which comprise DBCO‐modified antibodies DBCO‐αCD3 and DBCO‐αCD28. As shown in Figure 1, DBCO‐αCD3/28@MNs are designed to be applied to the skin overlaying the lymph nodes, enabling the transport of DBCO‐αCD3/28 to these regions. These antibodies bind to CD3 and CD28 receptors on T cells, activating them and labeling their surface with DBCO groups. In parallel, to label tumor cells in vivo, we prepared a dissolvable microneedle (IP10‐Az@MNs) loaded with azido sugar (Ac4ManNAz) and the T cell chemoattractant chemokine IP10. After insertion into the tumor site, Ac4ManNAz is released from the IP10‐Az@MNs and internalized by tumor cells, resulting in the generation of azide groups on the tumor surface through metabolic engineering. The IP10 released from IP10‐Az@MNs directs DBCO‐modified T cells to the tumor. Furthermore, the bioorthogonal reaction between the DBCO and N_3_ groups further enhances T cell accumulation in the tumor. Once there, the activated T cells engage with tumor cells and induce tumor‐specific cytotoxicity through the bioorthogonal click reaction. Our bioorthogonal‐based microneedle strategy offers a promising theoretical and experimental foundation for advancing T cell‐mediated antitumor immunity.

## 2 Results and Discussion

### 2.1 Fabrication and Characterization of MNs

**Figure 2:**
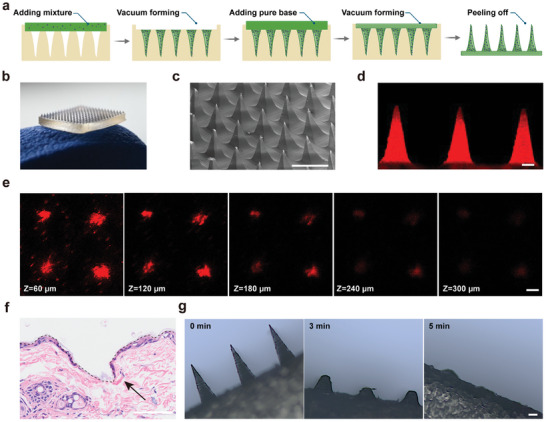
Fabrication and characterization of MNs. a) Schematic illustration of the MN preparation process. b) Photographic images of fabricated MNs. c) Representative SEM images of MNs, scale bar = 500 µm. d) CLSM image of PE‐αCD3 distribution in the needle tips of MNs, scale bar = 100 µm. e) Representative confocal laser scanning microscopy (CLSM) images of mouse skin detected at various depths following the administration of PE‐αCD3‐loaded MNs for 10 min, scale bar = 100 µm. f) H&E staining image of mouse skin after administration of MNs, scale bar = 50 µm. g) Dissolution images of blank MNs at various time intervals, scale bar = 50 µm.

Hyaluronic acid (HA), a natural component of the human body, exhibits notable versatility and good biocompatibility, making it an ideal material for the fabrication of dissolving microneedles.^[^ ^44^, ^45^ ^]^ The main preparation procedure of MNs and drug‐loaded MNs was illustrated in **Figure** **2**a. The MN dimensions were 8 mm × 8 mm, consisting of a 15 × 15 array of sharp, pyramidal shaped needles (Figure 2b,c). The needle height of the MNs was ≈500 µm, and the width of the base was ≈200 µm (Figure 2c; and Figure S1, Supporting Information). The sharpness of the MN tip was ≈3.2 µm (Figure S1, Supporting Information). To evaluate cargo distribution within the MNs, phycoerythrin (PE)‐labeled αCD3 was loaded into the MNs. As shown in Figure 2d; and Figures S2 and S3 (Supporting Information), PE‐labeled αCD3 was predominantly distributed within the needle tips. To penetrate the skin and deliver agents effectively, MNs require adequate mechanical strength. The mechanical strength of blank MNs made by only HA, IP10‐Az@MNs, and DBCO‐αCD3/28@MNs were tested using a universal testing machine. As shown in Figure S4 (Supporting Information), the mean of destructive force of single needles of the blank MNs, IP10‐Az@MNs, and DBCO‐αCD3/28@MNs were 1.037, 0.880, and 0.910 N, respectively, all of which were greater than the reported minimum skin insertion force (0.058 N).^[^ ^46^ ^]^ While the incorporation of agents, such as antibodies and IP10, slightly reduced the mechanical strength, the MNs remained robust enough to penetrate the skin without breaking. The successful insertion of MNs into the skin was verified by the deposition of PE‐labeled αCD3 within the epidermis at depths of ≈300 µm (Figure 2e) and the precise distribution of trypan blue spots at insertion sites (Figure S5, Supporting Information). Additionally, hematoxylin and eosin (H&E) stained images confirmed the formation of micropores due to skin rupture after MNs application (Figure 2f). To verify the dissolution characteristics, MNs were inserted into porcine skin. As shown in Figure 2g, the needles were completely dissolved within 5 min, indicating its excellent solubility. In vitro release profiles showed rapid IP10 release upon MN insertion into phosphate buffered saline (PBS), with over 90% released within 60 s (Figure S6, Supporting Information). The drug release behavior of MNs was further investigated using a skin tissue surrogate model designed to mimic the physiological skin environment.^[^ ^47^ ^]^ This model features a silicone rubber layer that replicates the stratum corneum and a gelatin‐based hydrogel that simulates the water‐rich viable epidermis.^[^ ^48^ ^]^ As shown in Figure S7a (Supporting Information), the IP10 release reached 60.38% after pressing the IP10‐loaded MNs into the skin tissue surrogates for 5 min, while nearly total release (93.64%) occurred at 60 min. The drug release performance of MNs was further investigated in vivo by inserting the Cy5‐labeled αCD3‐loaded MNs into the back skin of anesthetized mouse. Residual MNs were immersed in PBS at indicated time points and the remaining antibody was quantified using a spectrofluorometer. Similar to the in vitro results of using skin tissue surrogates, the release of antibodies reached 56.81% within 5 min and ≈90% within 60 min (Figure S7b, Supporting Information). This rapid release can be attributed to the dissolution and swelling properties of HA‐based microneedles. These results demonstrate that the MNs possess a well‐defined drug loading and release profile, making them suitable for subsequent in vitro and in vivo studies.

### 2.2 Bioorthogonal Labeling and Functional Characterization of DBCO‐αCD3/28@MNs and IP10‐Az@MNs

**Figure 3:**
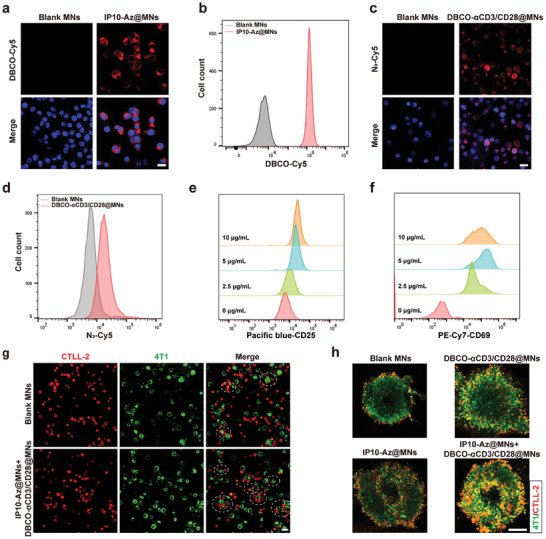
The bioorthogonal labeling performance of DBCO‐αCD3/28@MNs and IP10‐Az@MNs, and their effects on T cell recognition and infiltration. a) Representative CLSM images of 4T1 cells stained with DBCO‐Cy5 for 30 min after incubation with IP10‐Az@MNs. Scale bar = 20 µm. b) FSC analysis of 4T1 cells stained with DBCO‐Cy5 for 30 min after incubation with Ip10‐Az@MNs. c) The representative CLSM images of CTLL‐2 cells stained with N3‐Cy5 for 30 min postincubation with DBCO‐αCD3/28@MNs. Scale bar = 20 µm. d) Flow cytometry analyses of CTLL‐2 cells stained with N3‐Cy5 for 30 min postincubation with DBCO‐αCD3/28@MNs. e,f) FSC analysis of the expression of CD25, CD69 in CTLL‐2 cells after incubation with DBCO‐αCD3/28@MNs at different DBCO‐αCD3/28 doses for 3 days. g) The interaction between CTLL‐2 and 4T1 cells observed by CLSM imaging. The formed cell clusters of CTLL‐2 and 4T1 cells were indicated by the white circles (CTLL‐2 cells: red, labeled by Dil, 4T1 cells: green, labeled by Dio). Scale bar = 20 µm. h) CLSM images of the penetration of CTLL‐2 in 4T1 spheroids (CTLL‐2 cells: red, labeled by CD45‐PE, 4T1 cells: green, labeled by GFP). Scale bar = 100 µm.

Synthetic tetraacetyl N‐azidoacetyl‐D‐mannosamine (Ac4ManNAz) is a widely used cell‐labeling agent by incorporating azide groups (*─*N_3_) into cell surface glycans through metabolic glycoengineering.^[^ ^49^ ^]^ To verify the presence of N_3_ groups on tumor cells following treatment with IP10‐Az@MNs, we analyzed the fluorescence of cyanine5‐DBCO (DBCO‐Cy5) on the surface of live cells using confocal laser scanning microscopy (CLSM) and flow cytometry (FSC). As illustrated in **Figure** **3**a,b; and Figure S8 (Supporting Information), 4T1 cells treated with IP10‐Az@MNs effectively incorporated N_3_ groups into cell surface glycans, in contrast to 4T1 cells treated with blank MNs containing only HA, which served as a negative control. CD3 and CD28 antibodies are commonly used as agonist antibodies to induce T cell activation.^[^ ^50^, ^51^ ^]^ To activate T cells and incorporate DBCO groups onto the cell surface, αCD3 and αCD28 antibodies were conjugated with DBCO‐PEG‐NHS, thereby introducing the DBCO moieties (DBCO‐αCD3/28). Coomassie blue staining confirmed the successful conjugation of the antibodies to the DBCO‐PEG‐NHS linker. As shown in Figure S9 (Supporting Information), both DBCO‐αCD3 and DBCO‐αCD28 antibody‐polymer conjugates exhibited reduced electrophoretic mobility and appeared as diffuse bands on the gels compared to the native antibodies. A mixture of DBCO‐αCD3/28 antibodies and HA was then used to prepare DBCO‐αCD3/28@MNs for T cell labeling and activation. To investigate the conjugation of DBCO modified antibodies to the cell surface, DBCO‐αCD3/28@MNs‐treated T cells were incubated with sulfo‐cyanine 5‐azide (Cy5‐N_3_). These treated T cells exhibited a pronounced Cy5 fluorescence intensity on their surface, confirming successful DBCO conjugation (Figure 3c,d). The accumulation of DBCO‐αCD3/28 in lymph nodes is a crucial condition for T cell activation and labeling. To monitor in vivo distribution, αCD3/28‐PEG‐Cy5‐loaded hyaluronic acid microneedle (Cy5‐αCD3/CD28@MNs) were constructed. The Cy5 fluorescence of DBCO‐αCD3/28 was detected using an ex vivo imaging system. As shown in Figure S10a,b (Supporting Information), fluorescence signals appeared in lymph nodes 12 h after application, peaked at 24 h, and then gradually decreased. The number of Cy5‐positive cells in lymph nodes were counted by flow cytometry at different time points after Cy5‐αCD3/CD28@MNs application. Cy5 positive cells were highest 24 h after application, with an average of 36% of cells being Cy5 positive (Figure S10c, Supporting Information). The in vivo distribution showed that Cy5‐αCD3/CD28 was also mainly distributed in the kidney and liver except for lymph nodes (Figure S10d, Supporting Information), which is in agreement with results reported previously.^[^ ^43^ ^]^ We then evaluated the T cell activation potential of DBCO‐αCD3/28@MNs by assessing the surface expression of the activation markers CD69 and CD25. As shown in Figure 3e,f, CD69 and CD25 expression levels on T cells increased with higher concentrations of DBCO‐αCD3/28, although a slight decrease was observed at 10 µg mL^−1^. Over time, CD69 and CD25 expression levels peaked at 3 days and subsequently declined in DBCO‐αCD3/28@MNs‐treated T cells (Figure S11, Supporting Information). The results suggested that both the concentration and duration of DBCO‐αCD3/28 exposure are crucial for T cell activation, as excessively high concentrations or prolonged exposure may impair T cell effector functions. The storage stability of the encapsulated DBCO‐αCD3/28 was further tested by evaluating their capacity to activate T cells after being stored at 4 °C for 3 days. As shown in Figure S12 (Supporting Information), the antibody‐loaded MNs maintained good biological activity. In order to explore the influence of DBCO‐αCD3/28@MNs on T cell cytotoxicity, a cell counting kit‐8 (CCK‐8) assay was performed. As illustrated in Figure S13 (Supporting Information), DBCO‐αCD3/28@MNs exhibited no cytotoxicity on CTLL‐2 cells. Previous studies have shown that the costimulation of CD3 and CD28 receptors could promote T cell proliferation.^[^ ^52^, ^53^ ^]^ As expected, DBCO‐αCD3/28@MNs‐activated T cells proliferated faster than blank MNs‐treated T cells. The incorporation of two complementary bioorthogonal groups into distinct cellular populations enables their covalent linkage through click chemical reactions.^[^ ^54^ ^]^ Therefore, we further explored the targeting and binding ability of T cells to cancer cells after labeling them with two complementary bioorthogonal group. CLSM imaging revealed an increased number of T cell‐cancer cell clusters and larger cluster sizes compared to CTLL‐2 cells and 4T1 cells treated with MNs (Figure 3g; and Figure S14, Supporting Information). Considering the rapid chemical reactions induced by azido and DBCO groups, we further evaluated the interactions over time between DBCO‐αCD3/28@MNs treated CTLL‐2 cells and IP10‐Az@MNs treated 4T1 cells. As shown in Figure S15 (Supporting Information), the number of cell aggregates boosted gradually from 10 to 30 min compared to blank MNs treated group, and formed larger aggregates with longer incubation times. These results are in agreement with those reported by Li et al., which reported a significant increase in the cluster of N_3_‐T cells and BCN‐Raji cells after 10 min of coculture.^[^ ^30^ ^]^ Alternative to the tumor‐targeting capability, the effective infiltration of T cells into tumor tissues constitutes another critical factor in antitumor immunity.^[^ ^55^, ^56^ ^]^ To evaluate the potential of DBCO‐αCD3/28@MNs and IP10‐Az@MNs to enhance intratumoral T cell infiltration, we then conducted in vitro transwell invasion assays to measure the invasive capacity of T cells post‐treatment (Figure S16a, Supporting Information). As shown in Figure S16b (Supporting Information), treating T cells with DBCO‐αCD3/28@MNs or tumor cells with IP10‐Az@MNs both enhanced the infiltration capability of T cells compared to the group treated with MNs. Notably, the concurrent treatment of T cells and tumor cells exhibited the highest efficacy in promoting T cell invasion in vitro. The 3D spheroid cancer cell model represents an important tool to determine the ability of T cells to infiltrate tumors.^[^ ^57^, ^58^, ^59^, ^60^, ^61^ ^]^ Therefore, we further employed a 3D in vitro tumor model composed of GFP‐expressing 4T1 cells to study T cell tumor infiltration. As shown in Figure 3h and T cells treated with MNs were subject to penetrative obstruction and located only on the periphery of the tumor spheroid after 72 h of incubation with the tumorspheres. Remarkably, treatment with either DBCO‐αCD3/28@MNs or IP10‐Az@MNs alone resulted in a modest enhancement of T cell infiltration into the cell spheroids. However, the combination of both treatments proved to be more effective. To evaluate the kinetics of tumor spheroid invasion of DBCO labeled T cells, we utilized a 3D 4T1 spheroid invasion assay. The positions of CTLL‐2 cells in the 4T1 spheroid were tracked after labeling them with Dil and GFP. We discovered that the red fluorescence was initially aggregated at the cell spheroid edges within 4 h and then gradually moved toward the center in groups treated with the combination of IP10‐Az@MNs and DBCO‐αCD3/28@MNs. However, the red fluorescence continued to accumulate at the edges of the cell spheroids as incubation time increased in the blank MNs treated group (Figure S17, Supporting Information). We also investigated the infiltration of T cells treated with DBCO‐αCD3/28@MNs in the tumorspheres of different diameters, as guided by bioorthogonal mediations. As shown in Figure S18 (Supporting Information), T cells were primarily distributed in the center of tumorspheres ≈60 µm in diameter after cocultured with DBCO‐αCD3/28@MNs treated tumorsphere for 12 h, whereas they were only present at the edge of the tumorspheres, which are ≈150 µm in diameter. Interestingly, we observed that our SDMNS approach was able to promote T cell infiltration into the center of larger 3D tumorspheres (≈300 µm in diameter), although it takes longer compared to smaller tumor sizes (Figure 3h). These results suggested that for large tumors, repeated dosage or prolonged administration might be needed, but this comes with the potential for systemic toxicity and over‐activation of T cells. These results indicated that the bioorthogonal‐based microneedles targeting strategy has the potential to enhance the tumor‐targeting and infiltration capabilities of T cells.

### 2.3 DBCO‐αCD3/28@MNs and IP10‐Az@MNs Enhanced T Cell Cytotoxicity Against Tumor Cells In Vitro

**Figure 4:**
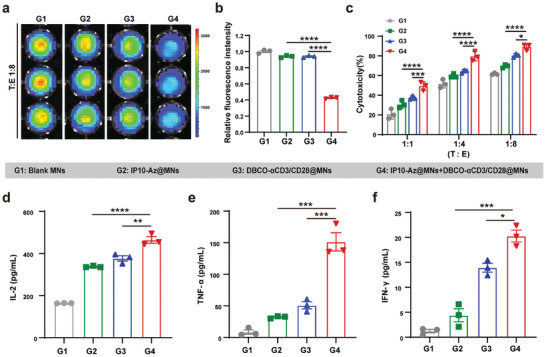
Cytotoxic effect of T cells against 4T1 cells after incubation with DBCO‐αCD3/28@MNs and IP10‐Az@MNs, respectively. a,b) Luminescence imaging assay a) and quantitative analysis b) (*n* = 3). c) LDH release from DBCO‐αCD3/28@MNs treated T cells against IP10‐Az@MNs treated 4T1 cells (*n* = 3). d–f) Cytokines secretion in DBCO‐αCD3/28@MNs treated T cells cocultured with IP10‐Az@MNs treated 4T1 cells (*n* = 3). *p < 0.05, **p < 0.01, ***p < 0.001, ****p < 0.0001.

To evaluate the cytotoxic effects of antitumor T cells mediated by DBCO‐αCD3/28@MNs and IP10‐Az@MNs, luciferase‐expressing 4T1 cells (4T1‐Luc cells, target) were treated with IP10‐Az@MNs and then incubated with DBCO‐αCD3/28@MNs‐treated T cells (effector) at an effector‐to‐target ratio of 1:1. As shown in **Figure** **4**a,b, the luminescence intensity of 4T1‐Luc cells decreased by over 50% in groups treated with the combination of IP10‐Az@MNs and DBCO‐αCD3/28@MNs, compared to the group treated with blank MNs alone. To further verify the enhanced cytotoxic effects resulting from the combined application of IP10‐Az@MNs and DBCO‐αCD3/28@MNs, tumor cells pretreated with IP10‐Az@MNs were coincubated with DBCO‐αCD3/28@MNs‐treated T cells at different effector‐to‐target ratios. Lactate dehydrogenase (LDH) is a cytosolic enzyme that can be rapidly released into the extracellular region upon cell membrane disruption. The release of LDH into the cell supernatant medium is widely used as an indicator of cell breakdown.^[^ ^62^ ^]^ Therefore, the amount of LDH released from damaged or lysed cells correlates with cytotoxic T cell activity.^[^ ^63^ ^]^ Therefore, LDH levels in the cell culture supernatant were quantified using an LDH release assay. As presented in Figure 4c, the amount of LDH released from G4 group exhibited a significant increase relative to the other groups. In addition, the cytotoxicity effect of T cells showed a dependency on the effector‐to‐target ratio. In addition, the CCK‐8 test and live/dead assays were further performed to illustrate the killing ability of tumor cells by T cells mediated by our SDMNS strategy. As shown in Figure S19 (Supporting Information), DBCO‐αCD3/28@MNs‐treated T cells cocultured with IP10‐Az@MNs‐treated tumor cells significantly suppressed the survival rate of tumor cells and promoted tumor cell death compared to those cocultured with blank MNs treated tumor cells. We also measured the cytokines released by T cells to further confirm the antitumor efficacy across different experimental groups. To detect cytokines secretion, T cells were added to the tumor cells at a 1:1 effector/target ratio and cocultured overnight. Cytokine release was measured using enzyme‐linked immunosorbent assay (ELISA) assay.^[^ ^64^, ^65^ ^]^ Obviously, the concentrations of interleukin‐2 (IL‐2), interferon‐γ (IFN‐γ), and tumor necrosis factor‐α (TNF‐α) in the culture supernatant coincubated with DBCO‐αCD3/28@MNs treated T cells and IP10‐Az@MNs treated tumor cells were markedly increased compared to other groups, aligning with the in vitro cytotoxicity results (Figure 4d–f). These results further illustrated that such bioorthogonal‐based microneedles had the efficacy to enhance T cell‐dependent antitumor immunity.

### 2.4 Enhanced Antitumor Effects of DBCO‐αCD3/28@MNs and IP10‐Az@MNs In Vivo

**Figure 5:**
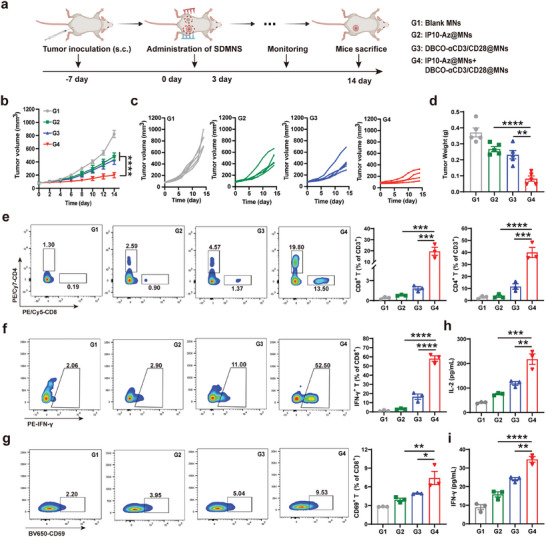
Antitumor efficacy of DBCO‐αCD3/28@MNs and IP10‐Az@MNs in a 4T1 xenograft mouse model. a) Schematic illustration of the treatment process. b,c) Average b) and individual c) tumor growth curves in 4T1 tumor‐bearing mice. d) Tumor weights post‐treatment (*n* = 5). e,f) Representative abundances of CD8^+^ T cells (gated on CD45^+^/CD3^+^/CD4^−^), CD4^+^ T cells (gated on CD45^+^/CD3^+^/CD8^−^) e), IFN‐γ^+^ T cells (gated on CD45^+^/CD3^+^/CD4^−^/ CD8^+^) f) in tumor tissues post‐treatment. The right panel displays the average abundances of corresponding lymphocyte subsets (*n* = 3). g) Representative abundances of CD69^+^ T cells (gated on live/CD45^+^/CD3^+^/CD4^−^/CD8^+^) in lymph node analyzed by FSC. The right panel displays the average abundances of corresponding lymphocyte subsets (*n* = 3). h,i) Plasma concentration of IL‐2 h) and IFN‐𝛾 i) in 4T1‐bearing mice at the end of treatment (*n* = 3). *p < 0.05, **p < 0.01, ***p < 0.001, ****p < 0.0001.

Based on the in vitro antitumor effects, we further carried out an in vivo experiment to evaluate the therapeutic effect in 4T1 tumor‐bearing mice. Once tumors reached ≈80 mm^3^, the mice were randomly assigned to four groups (*n* = 5 per group), including blank MNs (G1), IP10‐Az@MNs (G2), DBCO‐αCD3/28@MNs (G3), and DBCO‐αCD3/28@MNs + IP10‐Az@MNs (G4). Tumor‐draining lymph nodes (TDLNs) serve as first sites for draining the tumor, playing essential roles in initiating antitumor immune responses.^[^ ^66^, ^67^ ^]^ However, the function of tumor antigen‐specific T cells is suppressed due to the immunosuppressive microenvironment in TDLNs during tumor progression.^[^ ^68^ ^]^ Study has demonstrated that tumor antigen‐specific T cells existed not only in TDLNs but also in nondraining lymph nodes (NDLNs).^[^ ^69^ ^]^ Okamura et al. found that tumor‐reactive effector T cells in distant lymph nodes from a primary tumor may serve as cell sources for adoptive cell therapy, if they can be expanded in vitro.^[^ ^70^ ^]^ Resecting TDLNs does not affect tumor recurrence, but impaired systemic immunity can accelerate it, indicating the role of NDLNs in the antitumor immune response.^[^ ^69^ ^]^ TDLNs were reported at the contralateral side of the primary tumor.^[^ ^71^ ^]^ Therefore, the skin over the inguinal lymph node opposite the tumor was selected for the application of DBCO‐αCD3/28@MNs, while the tumor area was used for the application of IP10‐Az@MNs. The therapy schedule was showed in **Figure** **5**a. The tumor growth curve indicated that both IP10‐Az@MNs or DBCO‐αCD3/28@MNs treatment inhibited tumor growth, with the combination of both treatments being even more effective (Figure 5b,c; and Figure S20, Supporting Information). Consistent with the tumor growth curve and images after treatment, the histogram of tumor mass significantly exhibited a significant reduction in the DBCO‐αCD3/28@MNs + IP10‐Az@MNs groups, indicating the remarkable tumor suppression capability of our bioorthogonal‐based microneedles targeting strategy (Figure 5d). To assess the immunostimulatory impact of the combined treatment of DBCO‐αCD3/28@MNs with IP10‐Az@MNs, the tumor‐infiltrating T cells after treatment were collected and subsequently analyzed by FCS. The cell gating strategy used for analysis of tumor‐infiltrating CD8^+^, CD4^+^ T cells was displayed in Figure S21 (Supporting Information). CD8^+^ T cells were gated as CD45^+^/ CD3^+^/CD4^−^/CD8^+^ and CD4^+^ T cells were gated as CD45^+^/CD3^+^/CD8^−^/CD4^+^. The proportions of CD8^+^ and CD4^+^ T cells exhibited a modest increase in the treatment groups receiving either DBCO‐αCD3/28@MNs or IP10‐Az@MNs alone, as compared to the group treated with MNs. In contrast, the administration of DBCO‐αCD3/28@MNs in conjunction with IP10‐Az@MNs markedly augmented the tumor infiltration of CD8+ and CD4+ T cells (Figure 5e). We further performed immunohistochemistry (IHC) staining to detect CD8^+^ and CD4^+^ T cells in 4T1 tumor tissues. Consistent with FCS analysis, DBCO‐αCD3/28@MNs + IP10‐Az@MNs treatment led to a greater increase in the number of CD8^+^ and CD4^+^ T cells in tumor tissues compared to other groups (Figure S22, Supporting Information). Given the crucial role of tumor‐infiltrating CD8^+^ T cells in mediating immune cytotoxicity, their immunophenotype were subjected to further evaluation.^[^ ^72^ ^]^ Interferon‐γ (IFN‐γ) is a well‐characterized cytokine produced by activated T cells and plays a critical role in antitumor immunity.^[^ ^73^, ^74^ ^]^ Therefore, the proportion of IFN‐γ‐producing CD8^+^ T cells can serve as an indicator of tumor‐infiltrating T cell activation status. The combination treatment significantly elevated the proportion of IFN‐γ^+^ CD8^+^ T cells (gated on CD45^+^/CD3^+^/CD4^−^) (Figure 5f). DBCO‐αCD3/28@MNs were administered to the skin overlying the lymph node with the purpose of releasing DBCO‐αCD3/28 antibodies, aiming to augment T cell activation, while facilitate the incorporation of the DBCO functional motif into the T cells. Therefore, lymph nodes were harvested from 4T1 tumor‐bearing mice post‐treatment, and the quantity of activated T cells within these lymph nodes was subsequently analyzed. CD69 expression was examined as an indicator of early T cell activation.^[^ ^75^, ^76^ ^]^ To quantify T cell activation in lymph nodes, samples were gated on live/CD45^+^/CD3^+^/CD4^−^/CD8^+^/CD69^+^ T cells (Figure S23, Supporting Information). A significantly higher percentage of these cells in mice treated with DBCO‐αCD3/28@MNs + IP10‐Az@MNs compared to other groups, indicating the combinational microneedles strategy could effectively enhances CD8^+^ T cells activation within lymph node (Figure 5g). As shown in Figure 5h,i; and Figure S24 (Supporting Information), the DBCO‐αCD3/28@MNs + IP10‐Az@MNs group presented the highest levels of IL‐12, IFN‐γ, and TNF‐α in comparison to the other groups. The treatments did not cause significant differences in mouse body weight, suggesting that the treatment was nontoxic (Figure S25, Supporting Information). Systemic administration of cytokines and immune checkpoint inhibitors are potent immunotherapeutics, but they might lead to severe dose‐limiting toxicity.^[^ ^77^ ^]^ Transdermal delivery of cytokines and ICIs dissolvable MNs was confirmed to mitigate the potential risk of immune‐related adverse events while maintaining the therapeutic effect.^[^ ^78^ ^]^ In the present study, we found that no obvious toxicity to major organs was observed though histological analysis following the administration of DBCO‐αCD3/28@MNs, IP10‐Az@MNs or their combination (Figure S26, Supporting Information). Additionally, serum biochemical indexes analysis and routine blood examination were performed. As shown in Figure S27 (Supporting Information), all routine blood parameters remained within normal limits, and no obvious differences were observed across all experimental groups. The key hepatic and renal function markers including uric acid, carbonyl diamide, aspartate aminotransferase, and alanine aminotransferase, showed negligible differences across all groups, as illustrated in Figure S28 (Supporting Information). These results suggested that the combination of DBCO‐αCD3/28@MNs with IP10‐Az@MNs effectively activated antitumor immunity by enhancing CD8^+^ T cell infiltration and activation. Additionally, the bioorthogonal‐based microneedles strategy demonstrated safety and did not cause toxicity in mice model.

### 2.5 Abscopal Effect of DBCO‐αCD3/28@MNs and IP10‐Az@MNs Mediated Antitumor Immunity

**Figure 6:**
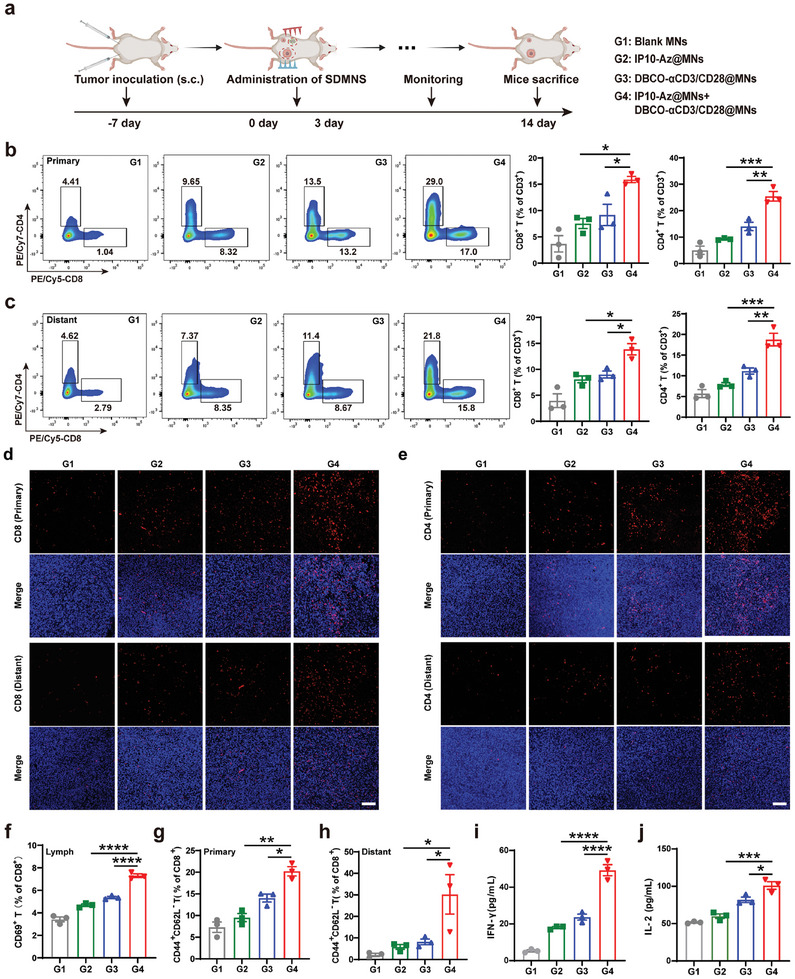
Abscopal effect and systemic antitumor immunity of DBCO‐αCD3/28@MNs and IP10‐Az@MNs in vivo. a) Schematic illustration of the treatment procedure. b,c) Representative abundances of CD8^+^ T cells (gated on CD45^+^/CD3^+^/CD4^−^), CD4^+^ T cells (gated on CD45^+^/CD3^+^/CD8^−^) in primary b) and distant c) tumor tissues post‐treatment. The right panel displays the average abundances of corresponding lymphocyte subsets (*n* = 3). d,e) Representative images of CD8^+^ T d) and CD4^+^ T e) cells staining in primary and distant tumor tissues after different treatments. Scale bar = 50 µm. f) Quantification of CD69^+^ T cells (gated on live/CD45^+^/CD3^+^/CD4^−^/CD8^+^) in lymph node analyzed by FSC. g,h) Quantification of CD44^+^ CD62L^−^ CD8^+^ T cells (gated on CD3^+^/CD4^−^) in primary g) and distant h) tumors analyzed by FSC (*n* = 3). i,j) Plasma concentration of IFN‐𝛾 i) and IL‐2 j) and in 4T1‐bearing mice at the end of treatment (*n* = 3). *p < 0.05, **p < 0.01, ***p < 0.001, ****p < 0.0001.

To further evaluate the impact of the bioorthogonal‐based microneedle strategy on T cells‐mediated antitumor immunity, we constructed a 4T1 bilateral tumor‐bearing mice model. The administration scheme for microneedles was illustrated in **Figure** **6**a. As shown in Figure S29a,b,e (Supporting Information), consistent with the previous results, the combination of DBCO‐αCD3/28@MNs with IP10‐Az@MNs significantly inhibited primary tumor growth compared to mice that were only treated with DBCO‐αCD3/28@MNs or IP10‐Az@MNs alone. Moreover, the antitumor efficacy elicited by the combination strategy against the untreated remote tumors was significant, suggesting that this effect may be attributed to the activation of systemic antitumor immunity (Figure S29c,d,f, Supporting Information). Consistently, the weights of primary and distant tumors in the DBCO‐αCD3/28@MNs + IP10‐Az@MNs group were also lower than those observed in either the DBCO‐αCD3/28@MNs or IP10‐Az@MNs groups (Figure S29g,h, Supporting Information). Inspired by the antitumor efficacy of DBCO‐αCD3/28@MNs and IP10‐Az@MNs combination treatment on distant tumors, we further examined the impact of these combination microneedles on the intratumoral infiltration of CD4^+^ and CD8^+^ T cells in 4T1 bilateral tumor‐bearing mice. FSC analysis showed a higher infiltration of CD4^+^ and CD8^+^ T cells in both primary and distant tumors compared to the DBCO‐αCD3/28@MNs or IP10‐Az@MNs groups (Figure 6b,c). The fluorescence staining results were in line with those of the FSC analysis, showing a significant increase in CD8^+^ and CD4^+^ T cells in primary and distant tumors in the DBCO‐αCD3/28@MNs and IP10‐Az@MNs treatment group (Figure 6d,e; and Figure S30, Supporting Information). In addition, the concomitant administration of DBCO‐αCD3/28@MNs and IP10‐Az@MNs resulted in a significant elevation in the proportion of IFN‐γ‐secreting CD8^+^ T cells within the primary and distant tumors, compared to the other experimental groups (Figure S31, Supporting Information). The combination of DBCO‐αCD3/28@MNs and IP10‐Az@MNs was observed to significantly increase the population of CD69^+^ T cells within the lymph nodes (Figure 6f). The generation of antigen‐specific memory lymphocytes is critical for establishing an effective and long‐lasting immune response against cancer.^[^ ^79^, ^80^ ^]^ Therefore, to validate the effect of immunological memory responses activated by DBCO‐αCD3/28@MNs and IP10‐Az@MNs, we conducted a FSC analysis to examine the effector memory T cells (T_EM_, CD3^+^ CD8^+^ CD44^+^ CD62L^−^) in the tumors and spleens across different groups. The cell gating strategy used for analysis of tumor‐infiltrating CD44^+^ CD62L^−^ CD8^+^ T cells (gated on CD3^+^/CD4^−^) was shown in Figure S32 (Supporting Information). A significant elevation in T_EM_ cell populations was observed in both primary and distant tumors in mice administered with DBCO‐αCD3/28@MNs combined with IP10‐Az@MNs, in comparison to those treated with either DBCO‐αCD3/28@MNs or IP10‐Az@MNs alone (Figure 6g,h). Interestingly, the percentage of T_EM_ cells in the G4 group in the distant tumors was much higher than that of the primary tumor. This difference might be attributed to the proximity of the DBCO‐αCD3/28@MNs insertion site to the TDLNs of the distant tumor. A Previous study demonstrated that the unilateral application of MNs on the dorsal area of mice skin could facilitate the cargo transport to the lymph nodes on both the ipsilateral and contralateral sides near to the administration site.^[^ ^43^ ^]^ Through intravital imaging, we found that, in addition to the ipsilateral lymph nodes, fluorescence also accumulated in the lymph nodes distal to the insertion site (Figure S33a, Supporting Information), which was consistent with the previous report.^[^ ^43^ ^]^ The fluorescence intensity in ipsilateral lymph nodes site was significantly higher than that distal lymph nodes at the time point 24 h after insertion. Even 72 h after the insertion, the lymph nodes on the ipsilateral side of the insertion sites still showed higher fluorescence (Figure S33b, Supporting Information), indicating the loaded cargo distribution might be not uniform in lymph nodes, but concentrated near insertion sites. In addition, the location of MNs insertion might affect the site of immunization.^[^ ^81^ ^]^ The spleens of mice treated with DBCO‐αCD3/28@MNs and IP10‐Az@MNs showed increased TEM cell frequency (Figure S34, Supporting Information). ELISA results indicated significantly higher serum levels of IFN‐γ, TNF‐α, and IL‐2 in the DBCO‐αCD3/28@MNs + IP10‐Az@MNs group compared to others (Figure 6i,j; and Figure S35, Supporting Information). To investigate the durability of the immune response elicited by the cotherapy of DBCO‐αCD3/28@MNs and IP10‐Az@MNs, the observation period following the final administration of MNs was extended to 30 days. At the end of the experiment, tumor tissues were harvested for immunofluorescent staining. The results suggested that the tumor‐infiltrating CD44^+^ CD62L^−^ cells persisted within the tumor tissues after the combined DBCO‐αCD3/28@MNs and IP10‐Az@MNs treatment (Figure S36, Supporting Information), indicating that this approach has the potential to induce durable antitumor immunity. In summary, these results indicated that the simultaneous administration of DBCO‐αCD3/28@MNs and IP10‐Az@MNs effectively inhibited tumor growth by increasing infiltrating cytotoxic CD8^+^ T cells at both primary and distant tumor sites and inducing long‐term immunological memory.

## 3 Conclusion

In this study, a spatially distributed microneedle system based on bioorthogonal reaction was developed to selectively label distinct cell types in vivo by orthogonally targeting azide and DBCO moieties. This multifunctional microneedle system comprised two distinct MNs, each serving a unique purpose and targeting a separate area. The DBCO‐αCD3/28@MNs were administered to lymph nodes, where they released DBCO‐αCD3/28 to activate T cells and label them with DBCO groups. To facilitate the targeted migration of activated T cells to the tumor site, IP10‐Az@MNs were engineered and applied locally at the tumor site. These microneedles facilitated the labeling of tumor cells with N_3_ groups through the release of Ac4ManNAz at the tumor site. Additionally, the tumor‐specific accumulation of activated T cells, induced by DBCO‐αCD3/28@MNs, was redirected by IP10 released from IP10‐Az@MNs and the bioorthogonal reaction between DBCO and N_3_ groups. Finally, the DBCO‐modified activated T cells were efficiently bound to the N_3_‐functionalized tumor cell surfaces, thereby effectively inhibiting primary and distant tumor growth by triggering robust antitumor immune responses and establishing immunological memory.

MNs are an advanced transdermal drug delivery system capable of creating numerous microchannels in the stratum corneum upon insertion, enabling effective topical delivery of therapeutic agents. Studies have demonstrated that MNs achieve higher drug distribution in tumors located beneath their insertion sites compared to systemic drug delivery methods, making them widely used for administering various anticancer drugs.^[^ ^82^ ^]^ MNs are particularly suitable for treating superficial and subdermal tumors due to their limited tissue penetration depth. They are currently being explored for applications in cancers, such as breast cancer, skin cancer, and oral esophageal cancer.^[^ ^83^ ^]^ Moreover, MNs can be customized to match the target site and the specific drugs being delivered. For larger and deeper superficial or subdermal tumors, MNs with adjustable penetration depths and sizes can facilitate targeted drug release within the tumor. For nonsuperficial tumors, dissolving or implantable MNs can be applied at surgical excision sites to prevent tumor recurrence. In terms of drug delivery, MNs effectively introduce therapeutic agents into the epidermis or dermis, allowing their transportation and distribution to superficial lymph nodes via the skin's lymphatic capillaries. To deliver agents effectively to lymph nodes in patients with tumors, the skin overlying superficial nodes—such as the cervical, axillary, and inguinal lymph nodes—may serve as ideal sites for MN insertion. This approach enhances lymphatic targeting and supports localized drug delivery for improved therapeutic outcomes.

Conventional CAR‐T therapy involves isolating T cells from patients and genetically modifying them with CARs ex vivo to specifically target cancer cells.^[^ ^84^ ^]^ However, antigen heterogeneity and the lack of tumor‐specific biomarkers on the surfaces of solid tumor cells hinder CAR efficiency.^[^ ^9^, ^10^ ^]^ Our SDMNS strategy differs from CAR‐T therapy by employing two distinct MNs to label, activate, and direct endogenous T cells to the tumor site. This approach enables specific binding to tumor cells through bioorthogonal chemistry, avoiding the need for genetic modification. Bioorthogonal reactions are characterized by their high efficiency and selectivity. By introducing bioorthogonal reaction groups onto the surface of T cells, these cells gain the ability to precisely target tumor cells labeled with complementary groups. This approach enhances the infiltration of activated T cells into tumor tissues and boosts their tumor‐killing efficacy. Additionally, this strategy bypasses the need to account for the expression of tumor‐specific antigens, offering a straightforward and versatile T cell immunotherapy approach with potential applicability for treating various solid tumors.

## 4 Experimental Section

### Materials

Hyaluronic acid (HA) with a molecular weight of 10 kDa was obtained from Bloomage Freda Biopharm Co., Ltd. (Shandong, China). Dil and Dio were purchased from Invitrogen (USA). Anti‐CD3 (cat. no. BE0001‐1, clone: 145‐2C11), anti‐CD28 (cat. no. BE0015‐1, clone: 37.51) antibody were purchased from bioxcell. Recombinant mouse IP10 protein was purchased from absin. PE anti‐ CD3 antibody (Cat: 100 205, clone: 17A2) was purchased from biolegend. DBCO‐PEG 5000‐NHS was purchased from Xi'an ruxi biological technology Co., Ltd. (Xi'an, China). Ac4ManNAz and DBCO‐Cy5 was purchased from MCE (Beijing, China). N_3_‐Cy5 was purchased from Glpbio (USA). Staining antibodies including anti‐CD45 (Cat: 103 108), anti‐CD3 (Cat: 100 214), anti‐CD‐4 (Cat: 100 422), anti‐CD8 (Cat: 100 710), anti‐CD25 (Cat: 102 022), anti‐CD69 (Cat: 104 512), anti‐IFN‐γ (Cat: 505 808), anti‐CD44 (Cat: 103 012), anti‐CD62 (Cat: 104 405), fixable viability kit (Cat: 423 101) were purchased from biolegend. ELISA kit of IFN‐γ, IL‐2, and TNF‐α were purchased from proteintech (Wuhan, China).

### Cell Lines and Animals

4T1, 4T1‐Luc (luciferase‐expressing 4T1) breast cancer cells, and CTLL‐2 were obtained from cell bank of Chinese Academy of Science (Shanghai, China). They were cultured in RPMI‐1640 medium (Giboco) with 10% v/v fetal bovine serum (FBS, BI) and 1% penicillin–streptomycin (BI) at 37 °C with 5% CO_2_. Cells were cryopreserved and thawed as previously described.^[^ ^85^ ^]^ All animal experiments followed the Animal Welfare Ethics Committee (AWEC) guidelines at the Tenth Affiliated Hospital of Southern Medical University (approval number, IACUC‐AWEC‐202301105).

### Preparation of Microneedles

The blank MNs: 0.1 g HA (*M*_w_ = 10 kDa) was dissolved in 1 mL of ultrapure water. The HA solution was added to the polydimethylsiloxane (PDMS) microneedle mold and vacuumed for 5 min, repeating 5 times until all micropores were filled. Then the PDSM mold was dried overnight at 37 °C. After drying, carefully peel MNs from the PDMS mold and store them in the dryer place.

DBCO‐αCD3/28@MNs: the anti‐CD3 and anti‐CD28 antibodies were diluted in PBS and then centrifuged at 4500 g using an ultrafiltration centrifuge tube (10 kDa) to concentrate them. DBCO‐PEG 5000‐NHS linker was added to the above antibody solution, incubating overnight at 4 °C. After overnight reaction, the mixture was then centrifuged to remove the free DBCO linker. To obtain the DBCO‐αCD3/28@MNs, a 60 µL aliquot of well‐mixed aqueous solutions comprising DSPE‐PEG–NHS–αCD3 (0.6 mg mL^−1^), DSPE‐PEG–NHS–αCD28 (0.6 mg mL^−1^), and HA solution was added to the PDMS mold. The mold was subsequently subjected to a vacuum at a negative pressure of −0.07 MPa to force the mixed solution into the bottom of the PDMS mold cavities. After 2 h of drying time, the HA solution was added onto the PDMS mold and leaving it to dry overnight at 37 °C. DBCO‐αCD3/28@MNs was carefully detached from PDMS mold after complete desiccation. Each DBCO‐αCD3/28@MNs contained 36 µg DSPE‐PEG–NHS–αCD3 and 36 µg DSPE‐PEG–NHS–αCD28.

IP10‐Az@MNs: A 60 µL aliquot of well‐mixed aqueous solution comprising IP10 (0.6 mg mL^−1^), Ac4ManNAz (2.4 mg mL^−1^), and HA solution was added to the PDMS mold. IP10‐Az@MNs were prepared according to the DBCO‐αCD3/28@MNs. Each IP10‐Az@MNs contained 36 µg IP10 and 144 µg Ac4ManNAz.

### Drug Releasing Profile Assay of MNs

In vitro release studies were performed using PBS (pH 7.4) and a skin tissue model established by Makvandi et al. as the release medium.^[^ ^48^ ^]^ The skin tissue model was prepared as described previously and was divided into two steps.^[^ ^48^ ^]^ Briefly, for the topmost layer (mimicking human stratum corneum), parts A and B of Mold Star 20T resin were mixed and then placed in a vacuum drying oven to remove air bubbles. Subsequently, the resin was coated onto polymeric sheets using automatic film applicators to achieve a thickness of ≈20 µm. The film was cured in an oven at 80 °C for 4 h. For the hydrogel layer (which mimics viable epidermis and dermis), a mixture of NaCl (0.9 wt%) and acacia gum (1 wt%) was prepared in PBS with PVA (5 wt%, 13–23 kDa). Gelatin (27.6 wt%) was then added, and the mixture was heated between 40 and 50 °C until it dissolved. Subsequently, glycerol (5 wt%) and agarose (1 wt%) were introduced into the solution, glycerol at 5 wt% and agaroses at 1 wt% were added to the solution, and then the mixture was microwaved for 30 s until it boiled. For the skin tissue model, the mixture was promptly poured into a mold situated on the silicone film, achieving a thickness of 2 mm. Then, a freshly synthesized IP10‐loaded MNs were immersed in PBS or the skin tissue model, and incubated at 37 °C. IP10 released from MNs were tested using bradford assay, measuring supernatant absorbance at 595 nm. IP10 concentrations in samples were calculated with the standard curves prepared according to the manufacture's instruction (beyotime, China). For in vivo drug delivery, Cy5‐labeled αCD3‐loaded MNs were pierced into the dorsal skin of nude mice for 5 min and then left in place for different times before removal. Residue MN patches were redissolved, and drug concentrations were quantified by using a microplate spectrofluorometer.

### Mechanical Strength Test of MNs

The compression mode of a universal testing machine was employed to test the mechanical strength of MNs (Instron, USA). The MNs was placed flat on the lower grip of the mechanical tester and the sensor probe of the tester pressed the MNs at a rate of 0.1 mm min^−1^. The time recording started when the sensor touched the tip and stopped when the compression force reached 1 N.

### In Vivo Skin Insertion

To study skin insertion of MNs, the hair of dorsal skin in BALB/c mice was gently removed. The MNs were inserted vertically into the skin with a thumb pressing for 5 min and left in place for 5 min before removing. Then, the skin was stained with 1% Taipan blue for 10 min. After excess dye was wiped off, the blue dot matrix was photographed by a stereomicroscope. The ruptured skin area was fixed with paraformaldehyde (4%) for 30 min, then embedded in paraffin for H&E staining. To study the depth of antibody penetration, PE‐αCD3‐loaded MNs were prepared and then punctured into the dorsal skin of mice for 10 min. After removing PE‐αCD3‐loaded MNs, depth of penetration was observed by Z‐stack series scanning of CLSM (Carl Zeiss, Germany).

### Bioorthogonal Labeling of MNs

To determine the labeling efficiency of DBCO groups on murine CTLL‐2 cells, DBCO‐αCD3/28@MNs were freshly dissolved into 1 mL cells culture medium and then incubated with CTLL‐2 cells overnight. The cells were subjected to centrifugation and resuspended in PBS with N_3_‐Cy5 for 30 min, followed by analysis using FCS and CLSM. To incorporate azide groups into murine 4T1 cell surface, IP10‐Az@MNs were freshly dissolved into 1 mL cells culture medium and then incubated 4T1 cells for 48 h. After incubation, 4T1 cells were centrifuged and stained with DBCO‐Cy5 for 30 min before FCS and CLSM imaging.

### In Vitro Activation of T Cells

CTLL‐2 cells were cultured in a medium containing MNs or DBCO‐αCD3/28@MNs for 24 h. After centrifugation, the cells were treated with anti‐CD25 (pacific) antibodies and anti‐CD69 (PE) antibodies, respectively. The efficiency of T cells activation was detected by FCS.

### In Vitro Infiltration Capability of T Cells

Transwell invasion assays was employed to investigate the infiltration capability of T cells. In brief, GFP‐expressing 4T1 cells were placed into the lower chamber and incubated with MNs or IP10‐Az@MNs for 48 h. Simultaneously, CTLL‐2 cells were treated with MNs or DBCO‐αCD3/28@MNs for 48 h. Cold matrigel (BD Biosciences, China) was introduced into the upper chamber of transwell and incubated overnight at 37 °C. Once solidified, treated CTLL‐2 cells were cultured in the matrigel‐coated chamber and incubated with treated 4T1 cells seeded in upper chamber for 12 h. Migrated T cells were then collected from the lower chamber of transwell system and counted using FCS with anti‐CD45 (PE) antibodies.

### Targeting Assay

4T1 cells and CTLL‐2 cells were treated with IP10‐Az@MNs and DBCO‐αCD3/28@MNs separately for 48 h. Then both treated and untreated 4T1 and CTLL‐2 cells were stained with Dil and Dio, respectively, for 30 min. The stained 4T1 cells and CTLL‐2 cells were seeded onto a confocal dish at a ratio of 1:1 and incubated for 20 min at 37 °C. The targeting of treated CTLL‐2 cells to 4T1 cells was analyzed using CLSM imaging.

### Cytotoxicity Assay of T Cells

The CTLL‐2 cells were treated with DBCO‐αCD3/28@MNs for 72 h. The 4T1‐Luc cells were subjected to IP10‐Az@MNs treatment for a duration of 48 h, after which they were placed in 96‐well plates (1 × 10^5^ cells well^−1^). Stimulated T cells were then introduced at an effector: target cell (CTLL‐2 cells and 4T1 cells) ratio of 1:1, 1:4, and 1:8. Then the coculture supernatants were harvested by centrifugation and measured immediately forLDH levels using LDH activity assay kit (Solarbio, China). The formula for calculation of cytotoxicity activity of T cells was as follows: Cytotoxicity % = [(coculture cells—T cell alone—tumor cells alone))/(maximum of target cell lysis—target cells alone)] × 100. Luminescence imaging assay was used to measure T cell cytotoxicity against 4T1 cells. Treated 4T1‐Luc cells were cultured overnight in 96‐well plates, then stimulated T cells were added at a 1:1 ratio for 4 h. The luminescence intensity of 4T1‐Luc cells was quantified utilizing the IVIS Spectrum Imaging System (PerkinElmer IVIS Spectrum).

### CCK‐8 Assay

The CTLL‐2 cells were treated with DBCO‐αCD3/28@MNs for 72 h. The 4T1 cells were subjected to IP10‐Az@MNs treatment for a duration of 48 h, after which they were placed in 96‐well plates (1 × 10^5^ cells well^−1^). Stimulated T cells were then introduced at an effector: target cell (CTLL‐2 cells and 4T1 cells) ratio of 1:1. After incubating for 24 h, 10 µL of CCK‐8 solution (MP Biomedicals, Irvine, CA) was added to the cells and incubated for 2 h. Cell viability was measured at OD450 nm by a microplate reader.

### Calcein‐AM/PI Assay

The CTLL‐2 cells were treated with DBCO‐αCD3/28@MNs for 72 h. The 4T1 cells were subjected to IP10‐Az@MNs treatment for a duration of 48 h, after which they were placed in a confocal small dish. Stimulated T cells were then introduced at an effector: target cell (CTLL‐2 cells and 4T1 cells) ratio of 1:1. Calcein‐AM and PI (beyotime biotechnnology, China) were applied to stain the living and dead cells after a 24 h incubation, and the cells were then imaged using a confocal laser scanning microscope.

### 3D Spheroid Cell Invasion Assay

GFP‐expressing 4T1 cells (5 × 10^3^ cells well^−1^) were seeded in low adhesion U‐shaped 96‐well plates (corning, USA) with cell culture medium containing MNs or IP10‐Az@MNs to form spheroids with different sizes. The CTLL‐2 cells were treated with DBCO‐αCD3/28@MNs. After 72 h, they were stained with anti‐CD45 (PE) antibodies or Dil dye and then coincubated with the 3D tumor sphere for varying times as indicated for each experiment. CLSM imaging was carried out to measure the distribution of CTLL‐2 cells into 3D tumor sphere.

### Cytokine Release

The serum concentrations of IL‐2, IFN‐γ, and TNF‐α were assessed by quantikine ELISA kits (proteintech, China) as directed by the manufacturer's instructions.

### Ex Vivo Imaging

The Cy5‐αCD3/CD28@MNs were pierced into the dorsal skin of nude mic. At various time intervals, the inguinal lymph nodes, liver, kidney, spleen, heart, and lungs of the mice were collected for imaging, and the fluorescence intensity was measured. The imaging of in vivo bioluminescence was performed with a small animal live imager (PerkinElmer, USA).

### In Vivo Antitumor Efficacy

4T1 subcutaneous tumor model was established by injecting 1 × 10^6^ 4T1 cells in 100 µL PBS into the backs of female BALB/c mice. When tumors averaged ≈80 mm^3^, mice were randomly divided into four groups: G1, blank MNs; G2, IP10‐Ac4ManNAz@MNs; G3, DBCO‐αCD3/CD28@MNs; G4, IP10‐Ac4ManNAz@MNs with DBCO‐αCD3/CD28@MNs. Treatments were administered on days 0 and 3. The body weight and tumor size and were monitored every 2 days, and tumor volume was calculated as follows: volume (mm^3^)  = (length × width^2^)/2 (mm^3^). Mice were sacrificed at day 14 and day 33, and the tumor tissues, lymph nodes, spleen, and main organs were collected for further analysis.

### Abscopal Antitumor Efficacy

To establish the bilateral subcutaneous model, 4T1 tumor cells were administered via subcutaneous injection into the right (1 × 10^6^ cells) and left flanks (1 × 10^5^ cells) of female BALB/c mice. Once tumors reached ≈80 mm^3^, mice were grouped and treated as previously described. Post‐treatment, the tumor tissues, lymph nodes, spleen, and main organs were collected for further analysis.

### In Vivo Analysis of Immune Cells

After the final treatment, the collected tumor tissues were sectioned into small pieces and digested with collagenase type IV (1 mg mL^−1^, Sigma‐Aldrich) and DNase I (50 µg mL^−1^, Roche) at 37 °C for a duration of 60 min. The obtained tumor suspensions, along with lymph nodes and spleen, were filtered to get single cells, counted and diluted to 1 × 10^7^ cells mL^−1^ in ice cold PBS with 2% FBS. Before staining, single cells were pretreated with an anti‐CD16/32 antibody to block FcRs. Then, cells were surface labeled for 20 min at 4 °C and permeabilized and fixed for 20 min using a fixation/permeabilization kit (BD Biosciences). To analyze intracellular cytokine levels, brefeldin A (BFA; Biolegend) was applied to cells for 5 h before surface staining. After washing, staining with surface markers, and overnight fixation, cells were detected through the CytoFLEX flow cytometer (Beckman, USA).

### Histological Analysis

Tissues were preserved in 4% paraformaldehyde for a day and then embedded in paraffin. The paraffin sections were deparaffinization and rehydration. The tissue sections were stained with standard hematoxylin and eosin (H&E).

### In Vivo Biosafe Evaluation

The safety of MNs, IP10‐Az@MNs, and DBCO‐αCD3/CD28@MNs was assessed through the body weight in mice, histopathological analysis of major organs, and biochemical analysis of serum. The main organs sections were subjected to H&E staining and observed using CLSM imaging (Zeiss, Germany).

### Statistical Analysis

All results are presented as mean ± SEM from at least three independent experiments, each conducted in triplicate. The differences among groups were analyzed using one‐way ANOVA or student's *t*‐tests by GraphPad Prism 9.0 software (GraphPad Software, La Jolla, USA).

## Conflict of Interest

The authors declare no conflict of interest.

## Supporting information



Supporting Information

## Data Availability

The data that support the findings of this study are available from the corresponding author upon reasonable request.
