# Supplementary material for: A Spatially Distributed Microneedle System for Bioorthogonal T Cell‐Guided Cancer Therapy
Source: Adv Sci (Weinh). 2025 Feb 8;12(13):2416841. doi: 10.1002/advs.202416841 (PMC11967824; doi:10.1002/advs.202416841)
Supplement: Supplementary file 1 — Supporting Information [file ADVS-12-2416841-s001.docx]

Supporting Information

A Spatially Distributed Microneedle System for Bioorthogonal T Cell-Guided Cancer Therapy

*Lanya Li, Fei Wang, Shushan Mo, Junyao Deng, Xueyi Wang, Jiacong Ai, Yingxian Xiao, Yan Zeng, Qishan Li, Yixin Zhang, Limin Cai*, Zhenhua Li**

***E-mail: clmdgsrmyy18@smu.edu.cn (L. C.)

zhenhuali@hbu.edu.cn (Z. L.)


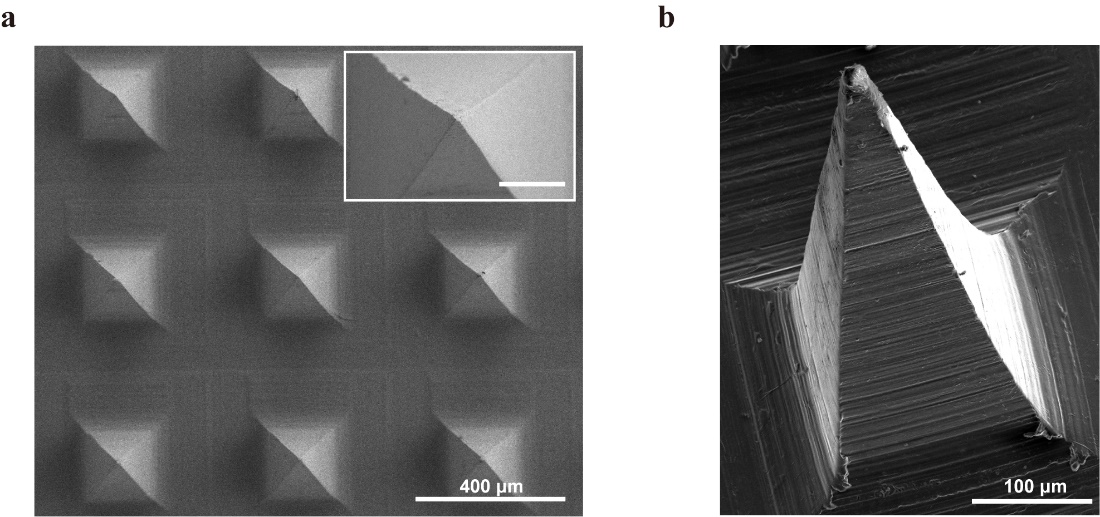


**Figure S1.** The representative SEM image of the top view (inset: MN tip, scale bar=50 μm) of MNs a) and the single MNs tip b).


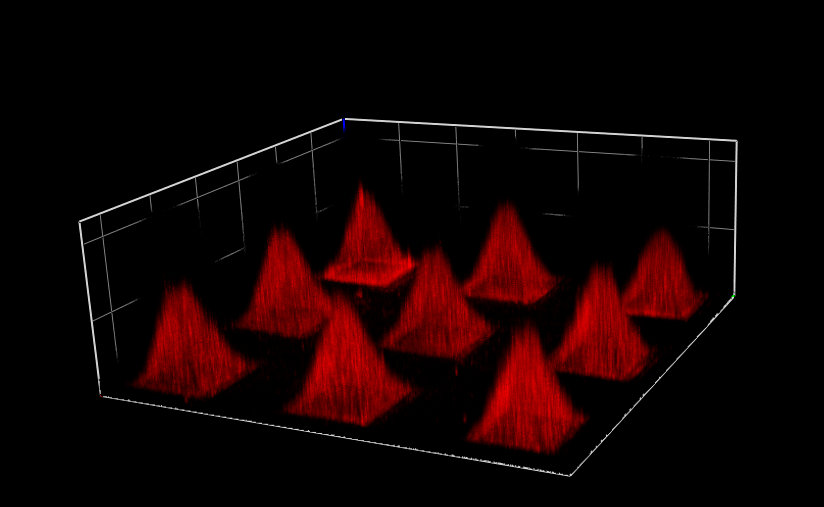


**Figure S2.** 3D reconstructed fluorescent image of MNs.


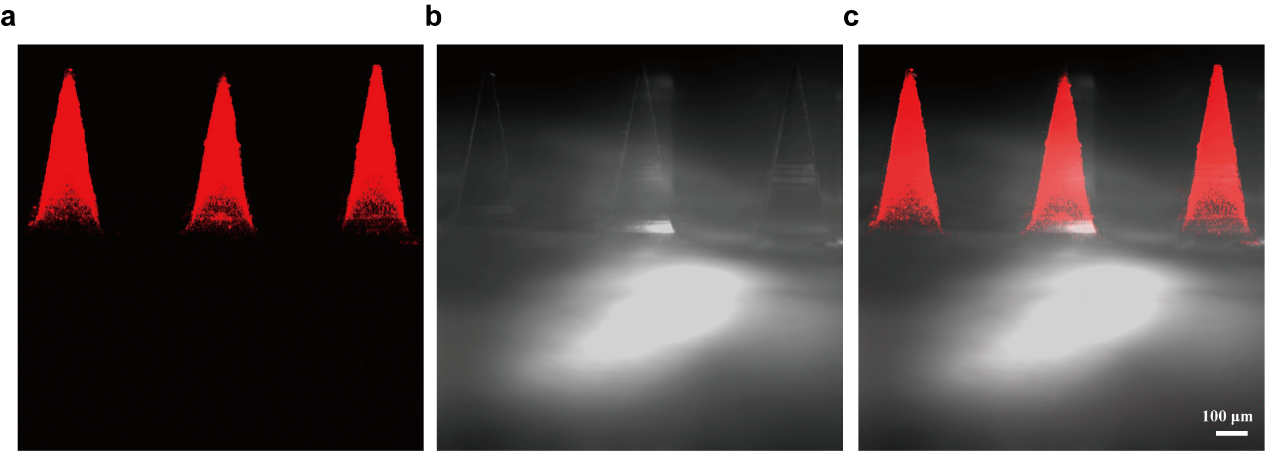


**Figure S3.** Fluorescent image a), bright field image b) and merge image c) of PE-αCD3 distribution in the needle tips of MNs, scale bar = 100 *μ*m.


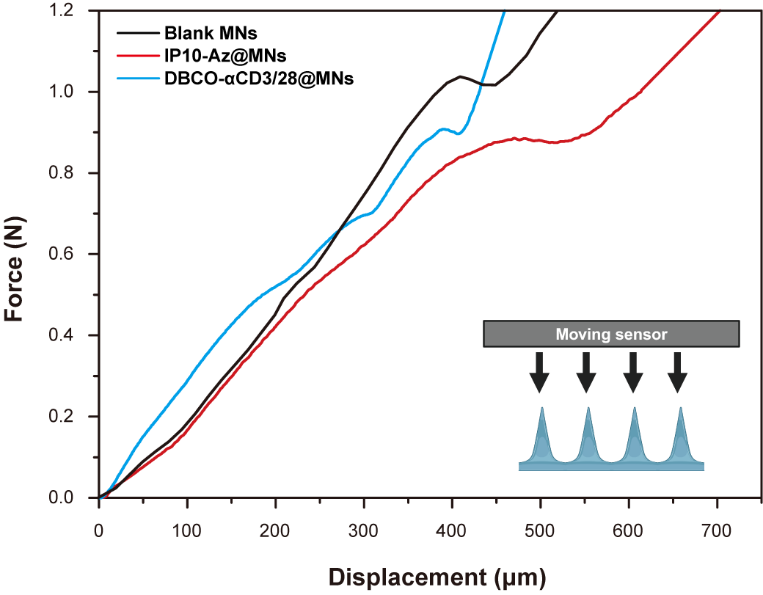


**Figure S4.** Force–displacement curves of blank MNs, IP10-Az@MNs and DBCO-αCD3/28@MNs.


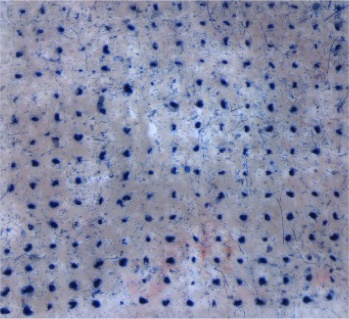


**Figure S5.** Image of the trypan blue stained mouse skin detected at the penetration of the MNs into the skin.


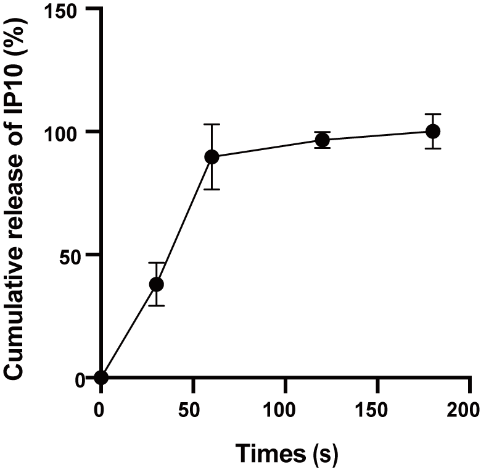


**Figure S6.** *In vitro* release profiles of IP10-loaded MNs in PBS (n=3).


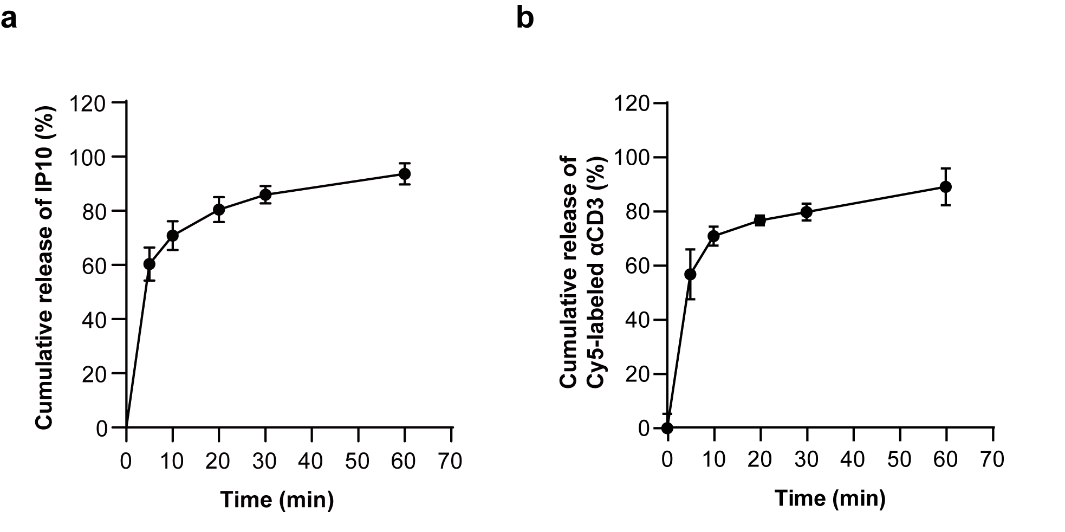


**Figure S7.** a) *In vitro* release profiles of IP10-loaded MNs in the skin tissue surrogates (n=3), b) *in vivo* release profiles of Cy5-labeled αCD3-loaded MNs (n=4).


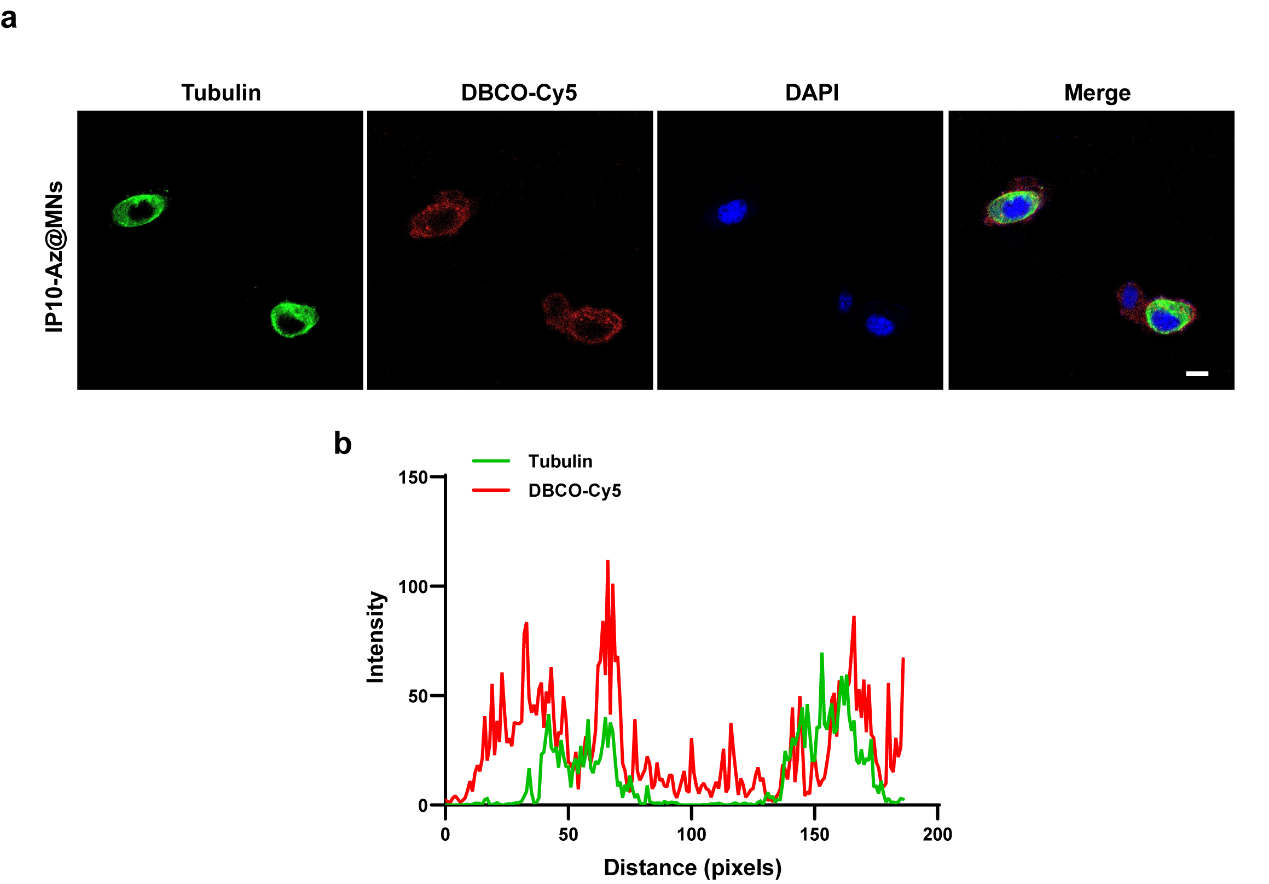


**Figure S8.** a) Co-localization images of DBCO-Cy5 (red) with tubulin (green) of 4T1 cells incubated with IP10-Az@MNs. Nuclei were stained with DAPI (blue). Merged panel show the co-localization of both signals. Scale bar=10 μm. b) Analysis of the colocalization of tubulin with DBCO-Cy5 in 4T1 cells.


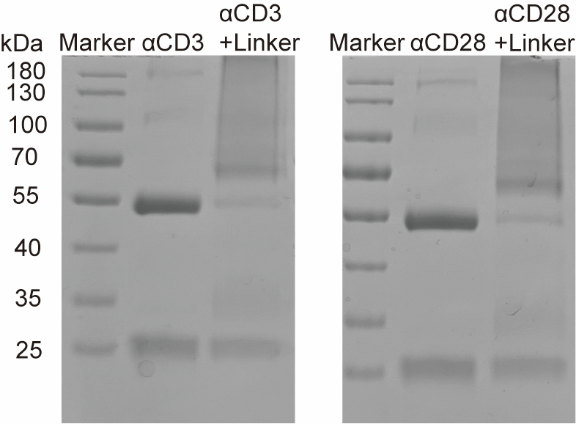


**Figure S9.** Coomassie blue staining analysis of the conjugation of anti-CD3 or anti- CD28 antibody and Linker.


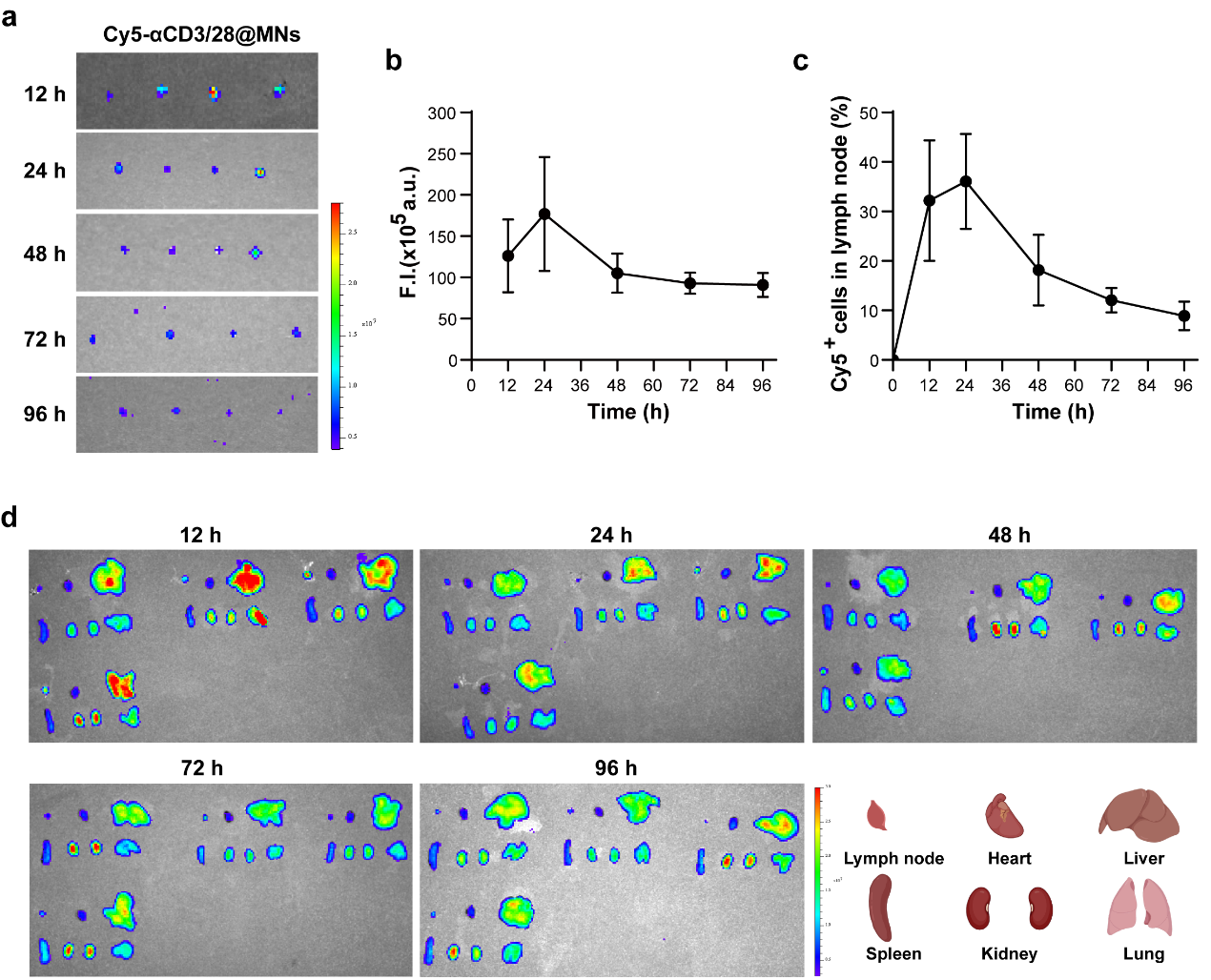


**Figure S10.** a) *Ex vivo* fluorescence imaging of ipsilateral inguinal lymph nodes near the sides of Cy5-αCD3/CD28@MNs insertion at different time points post-Cy5-αCD3/CD28@MNs application (n=4). b) Quantification of *ex vivo* fluorescence imaging of ipsilateral inguinal lymph nodes near the sides of Cy5-αCD3/CD28@MNs insertion (n=4). c) Flow cytometric analysis of Cy5^+^ cells in ipsilateral inguinal lymph nodes near the sides of Cy5-αCD3/CD28@MNs insertion from mice treated with Cy5-αCD3/CD28@MNs (n=4). d) *Ex vivo* fluorescence imaging of major organs at different time points post-Cy5-αCD3/CD28@MNs application (n=4).


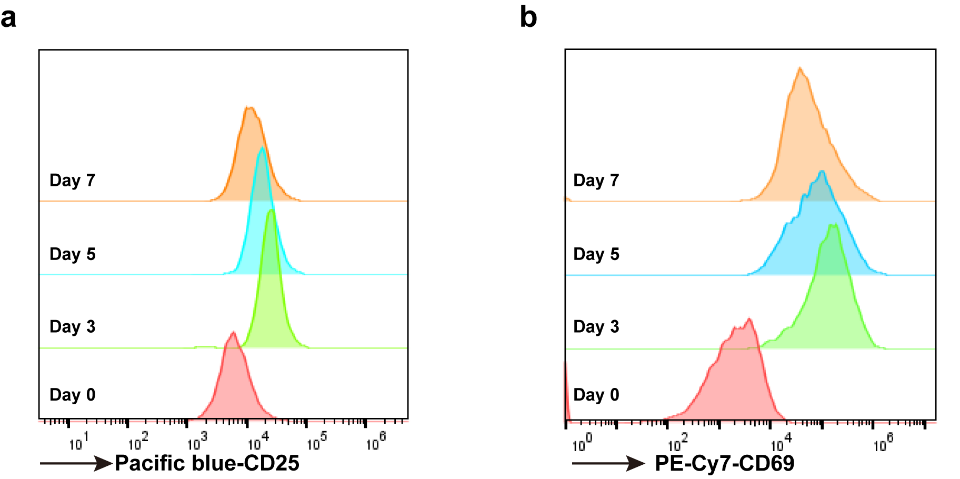


**Figure S11.** a, b) FSC analysis of the expression of CD25 a) and CD69 b) in CTLL-2 cells after incubated with DBCO-αCD3/28@MNs over different days.


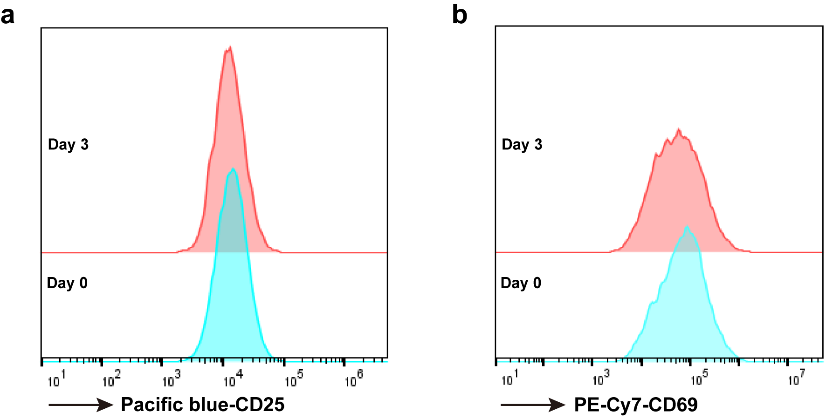


**Figure S12.** a, b) FSC analysis of the expression of CD25 a) and CD69 b) in CTLL-2 cells after incubation with DBCO-αCD3/28@MNs stored at 4 °C for 0 days and 3 days.


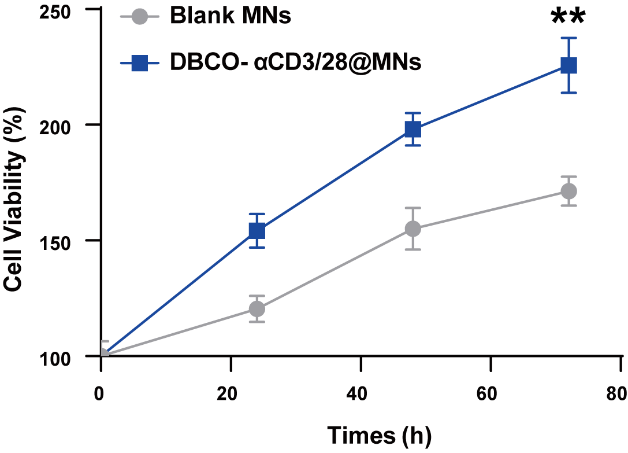


**Figure S13.** Effects of blank MNs and DBCO-αCD3/28@MNs on the viability rates of CTLL-2 cells (n=3). ***p*<0.01.


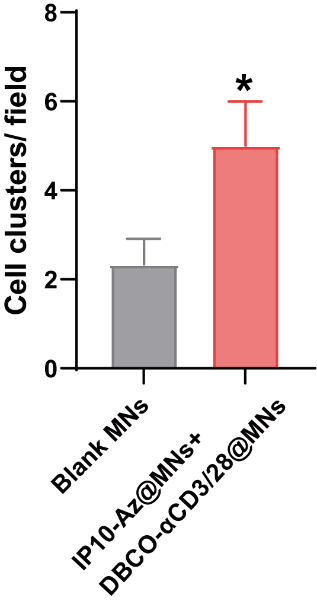


**Figure S14.** Quantification of the number of cell cluster in the co-culture of 4T1 and CTLL-2 cells with or without treatment by IP10-Az@MNs+DBCO-αCD3/28@MNs. For each cluster, a minimum of four cells comprising both T cells and tumor cells were enumerated (n=3 fields). **p*<0.05.


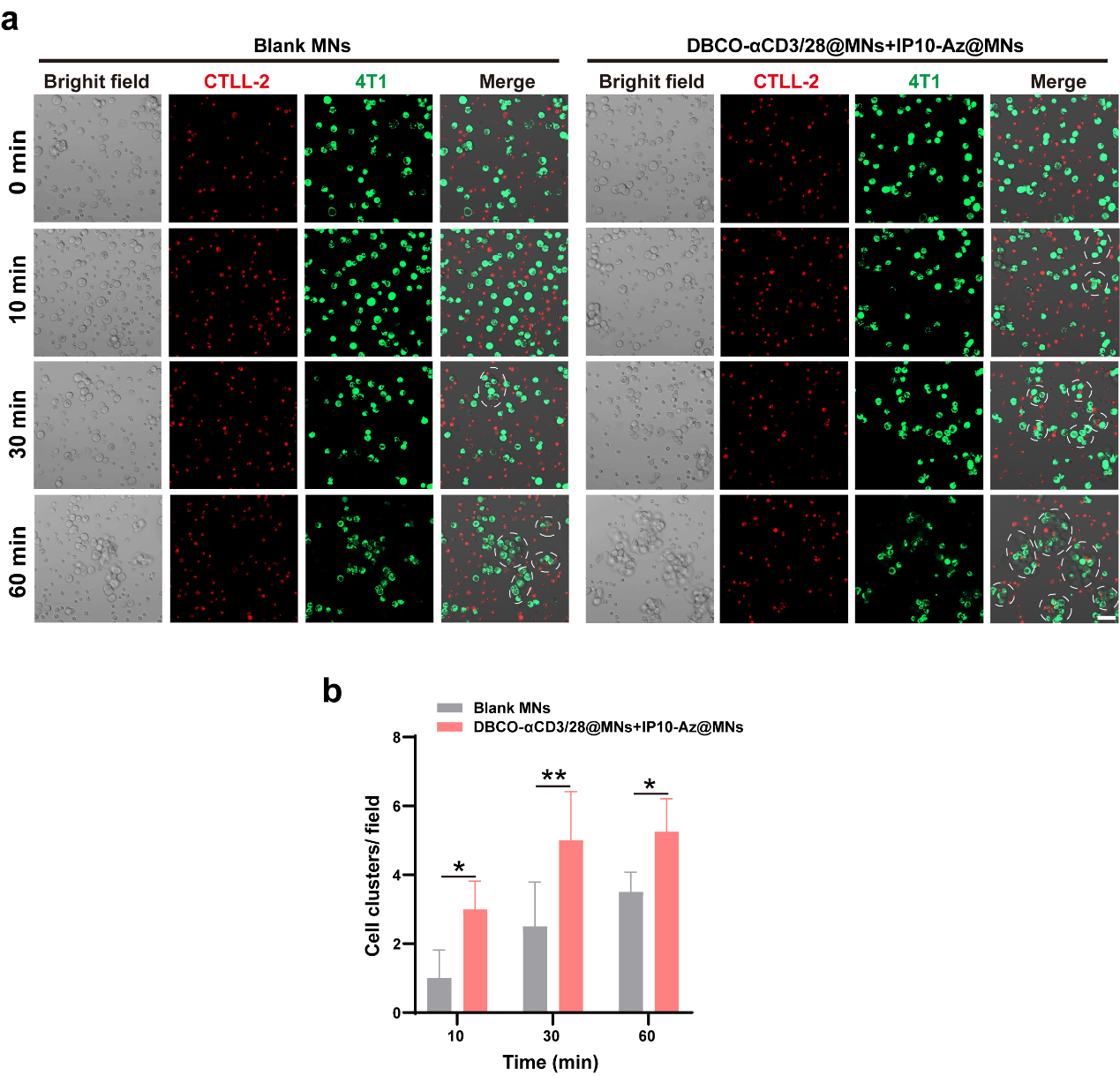


**Figure S15.** a) The interaction between CTLL-2 and 4T1 cells at different time (0 min, 10 min, 30 min, 60 min) observed by CLSM imaging. The formed cell clusters of CTLL-2 and 4T1 cells were indicated by the white circles (CTLL-2 cells: red, labeled by Dil, 4T1 cells: green, labeled by GFP). Scale bar=20 μm. b) Quantification of the number of cell cluster in the co-culture of 4T1 and CTLL-2 cells with treatment by blank MNs or IP10-Az@MNs+DBCO-αCD3/28@MNs. For each cluster, a minimum of four cells comprising both T cells and tumor cells were enumerated (n=3 fields). **p*<0.05, ***p* < 0.01.


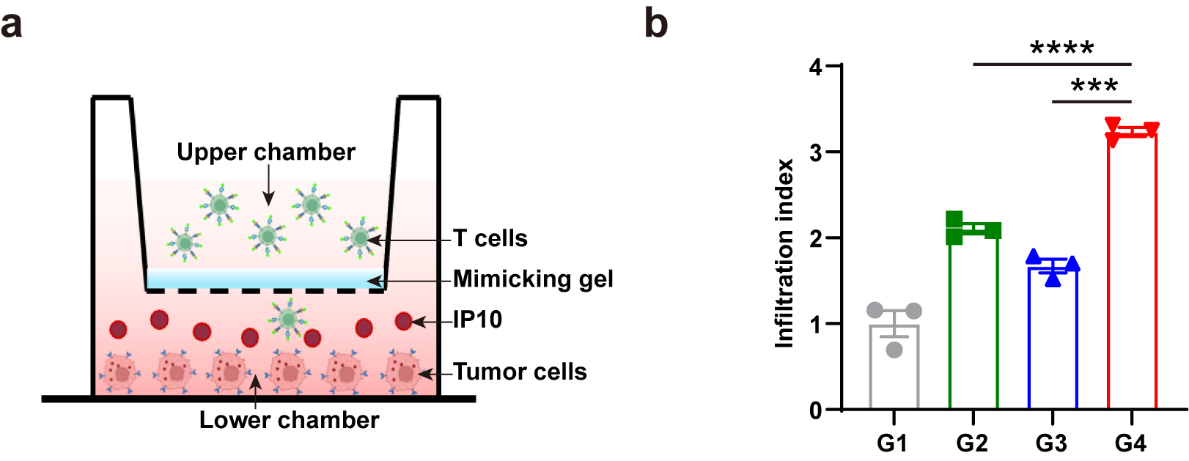


**Figure S16.** a) Diagram of the in vitro transwell invasion assays to assess T cell infiltration. b) Infiltration index of T cells following various treatments after a 12 h incubation (n =3). G1: Blank MNs, G2: IP10-Az@MNs, G3: DBCO-αCD3/28@MNs, G4: IP10-Az@MNs+DBCO-αCD3/28@MNs. ****p* < 0.001, *****p* < 0.0001.


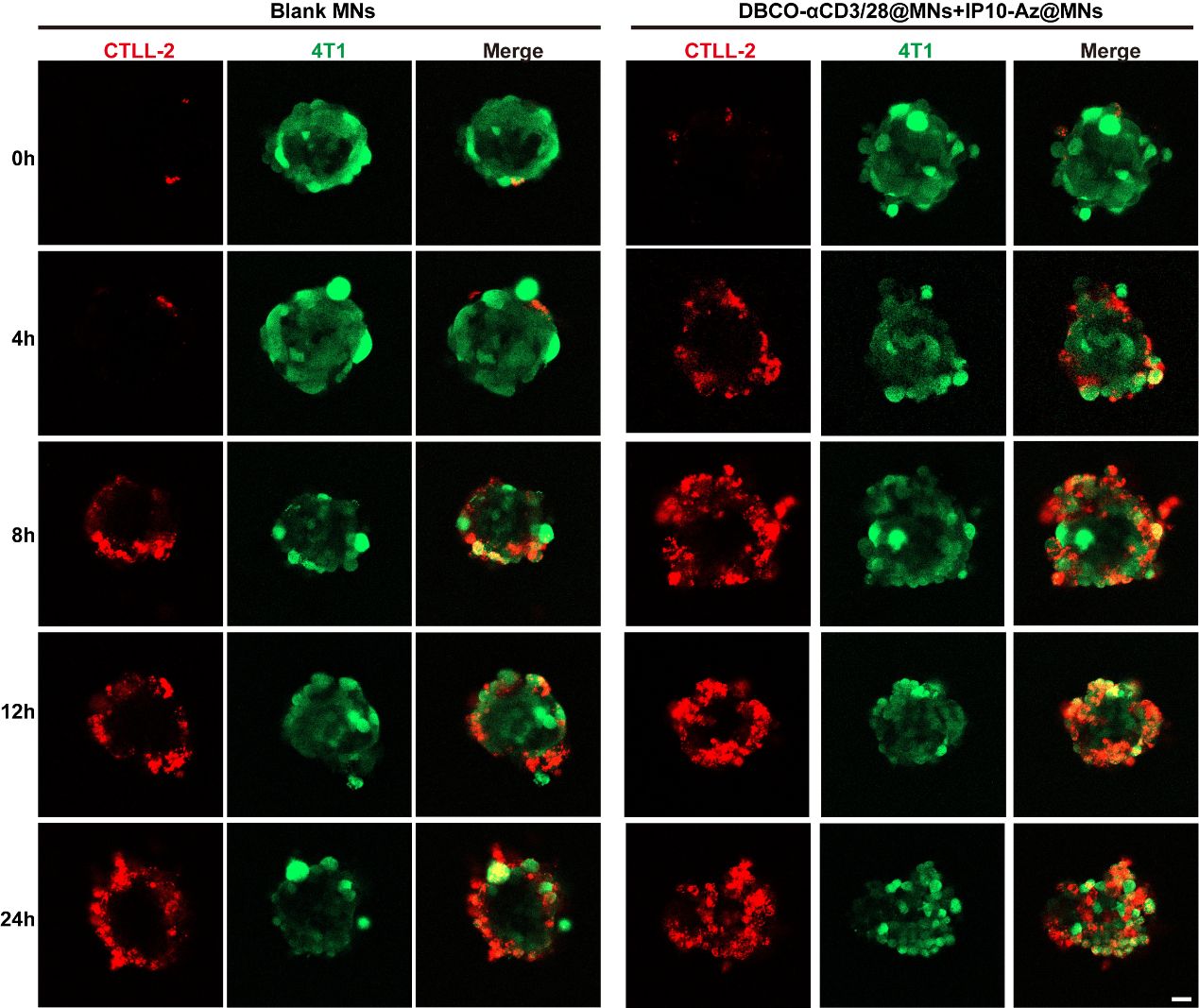


**Figure S17.** CLSM images of the penetration of CTLL-2 treated with DBCO-αCD3/28@MNs in 4T1 spheroids treated with IP10-Az@MNs after different time of incubation. (CTLL-2 cells: red, labeled by Dil, 4T1 cells: green, labeled by GFP). Scale bar=20 μm.


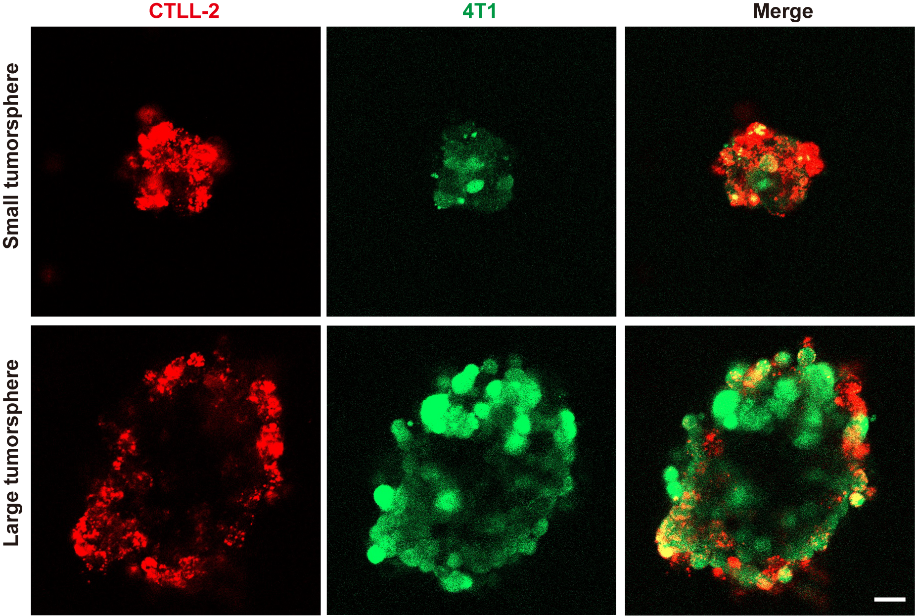


**Figure S18.** CLSM images of the penetration of CTLL-2 treated with DBCO-αCD3/28@MNs in 4T1 spheroids of different sizes treated with IP10-Az@MNs after 12 h of incubation. (CTLL-2 cells: red, labeled by Dil, 4T1 cells: green, labeled by GFP). Scale bar=20 μm.


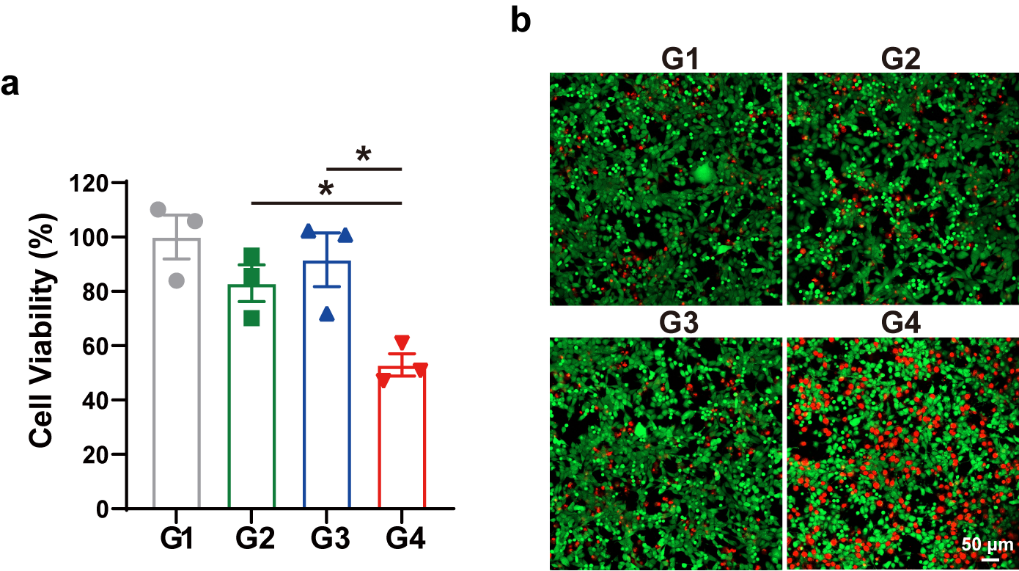


**Figure S19.** a) Survival rate of 4T1 cells after co-culturing with T cells under different treatments. **p* < 0.05. b) Live/dead staining images of 4T1 cells after co-cultured with CTLL-2 cells under different treatments. green: live cells, red: dead cells. Scale bar=50 μm. G1: Blank MNs, G2: IP10-Az@MNs, G3: DBCO-αCD3/28@MNs, G4: IP10-Az@MNs+DBCO-αCD3/28@MNs.


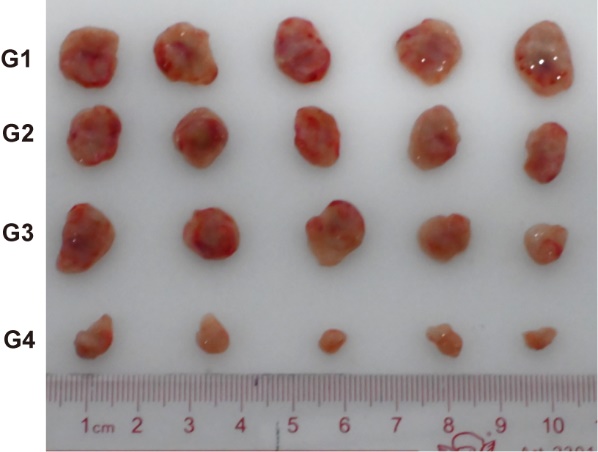


**Figure S20.** The picture of tumors from each group. G1: Blank MNs, G2: IP10-Az@MNs, G3: DBCO-αCD3/28@MNs, G4: IP10-Az@MNs+DBCO-αCD3/28@MNs.


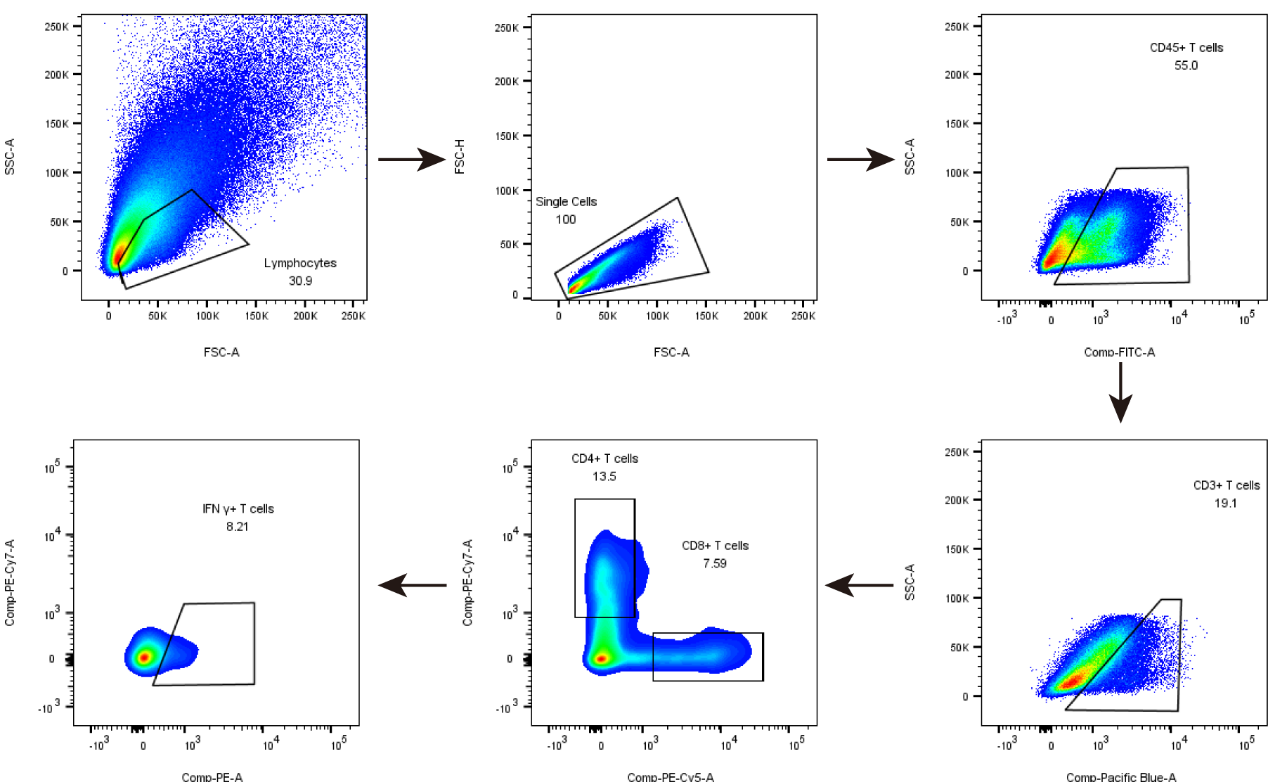


**Figure S21.** A loop-gate flow diagram was constructed to analyze and characterize the cluster of CD4^+^ (gated on CD45^+^/CD3^+^/CD8^−^), CD8^+^ (gated on CD45^+^/CD3^+^/CD4^−^) T cells, and IFN-γ^+^ CD8^+^ (gated on CD45^+^/CD3^+^/CD4^−^) T cells in the tumor.


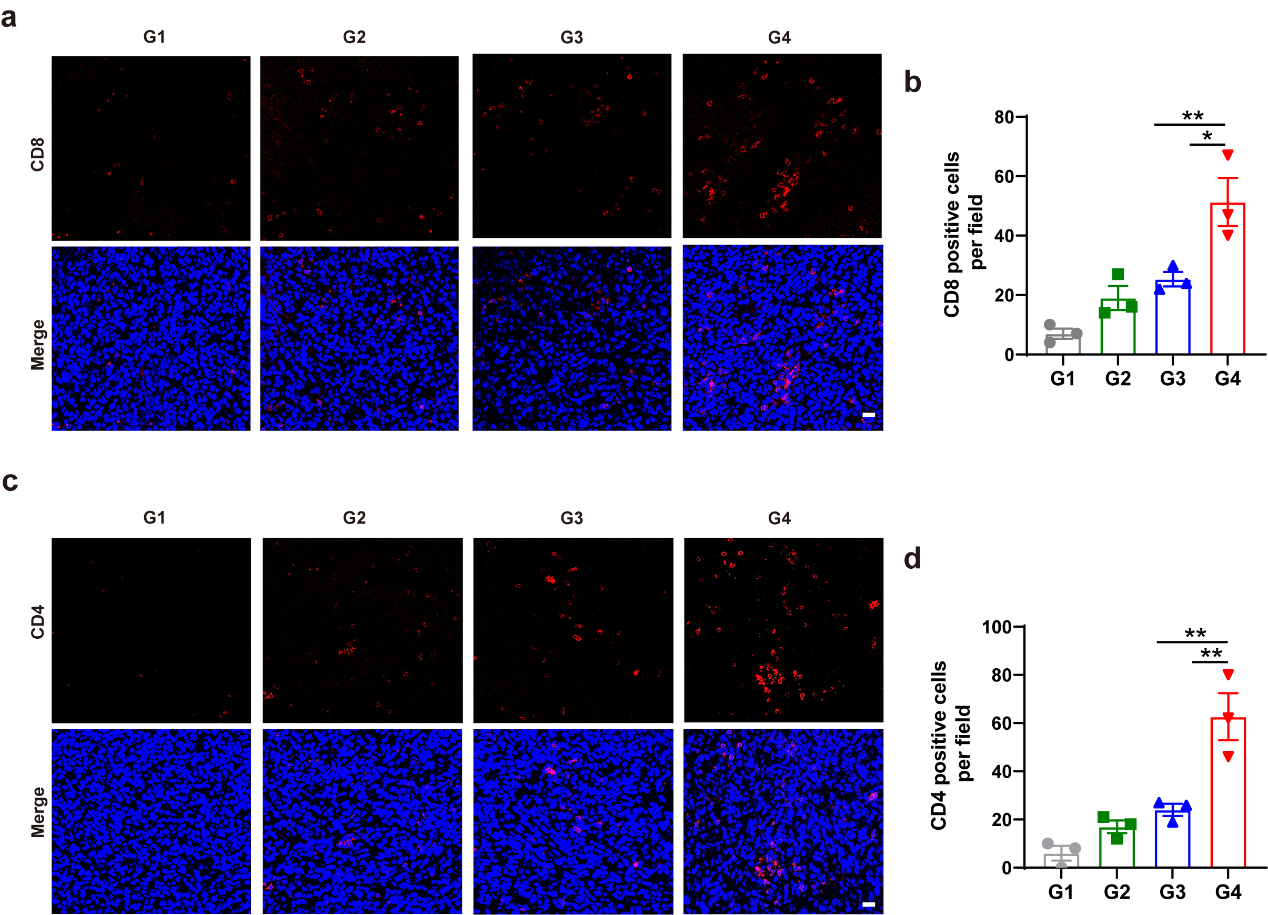


**Figure S22.** a, c) Representative images of CD4^+^ T a) and CD8^+^ T cells c) staining of tumor tissues after different treatments. b, d) Average number of CD8 b) and CD4 d) cells per field (n=3). G1: Blank MNs, G2: IP10-Az@MNs, G3: DBCO-αCD3/28@MNs, G4: IP10-Az@MNs+DBCO-αCD3/28@MNs. Scale bar=20 μm. * *p* < 0.05, ***p* < 0.01.


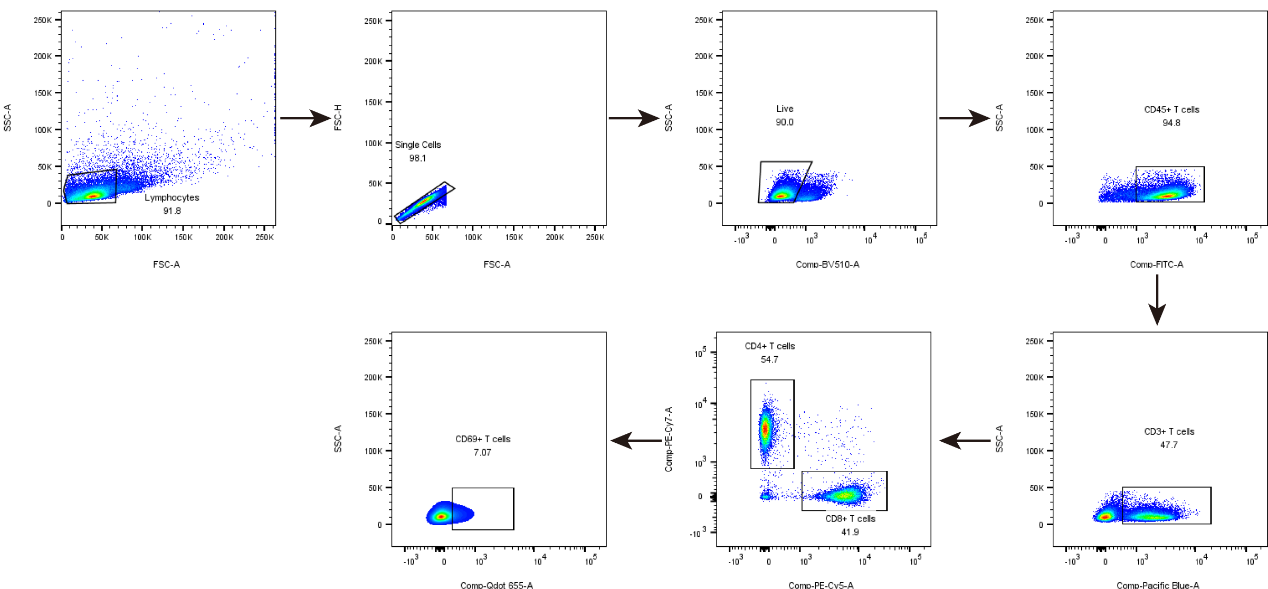


**Figure S23.** A loop-gate flow diagram was constructed to analyze and characterize the cluster of CD69^+^ CD8^+^ T cells (gated on live/CD45^+^/CD3^+^/CD4^−^) in the lymph nodes.


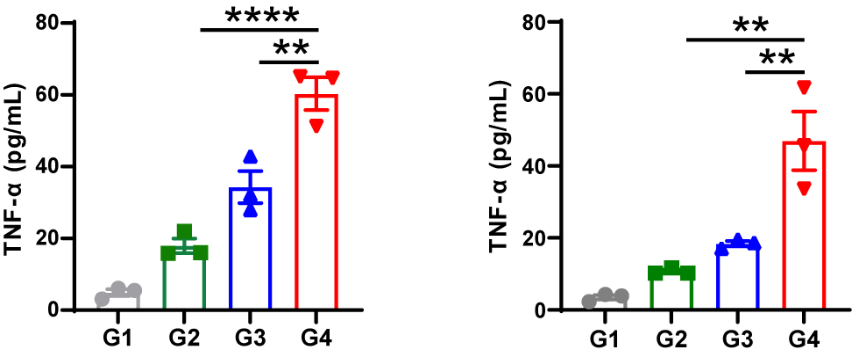


**Figure S24.** Plasma concentration of TNF-α in 4T1-bearing mice at the end of treatment (n=3). G1: Blank MNs, G2: IP10-Az@MNs, G3: DBCO-αCD3/28@MNs, G4: IP10-Az@MNs+ DBCO-αCD3/28@MNs. ***p* < 0.01.

**Figure S25.** Body weight curves of mice in different treatment groups within treatment (n=5). G1: Blank MNs, G2: IP10-Az@MNs, G3: DBCO-αCD3/28@MNs, G4: IP10-Az@MNs+DBCO-αCD3/28@MNs.


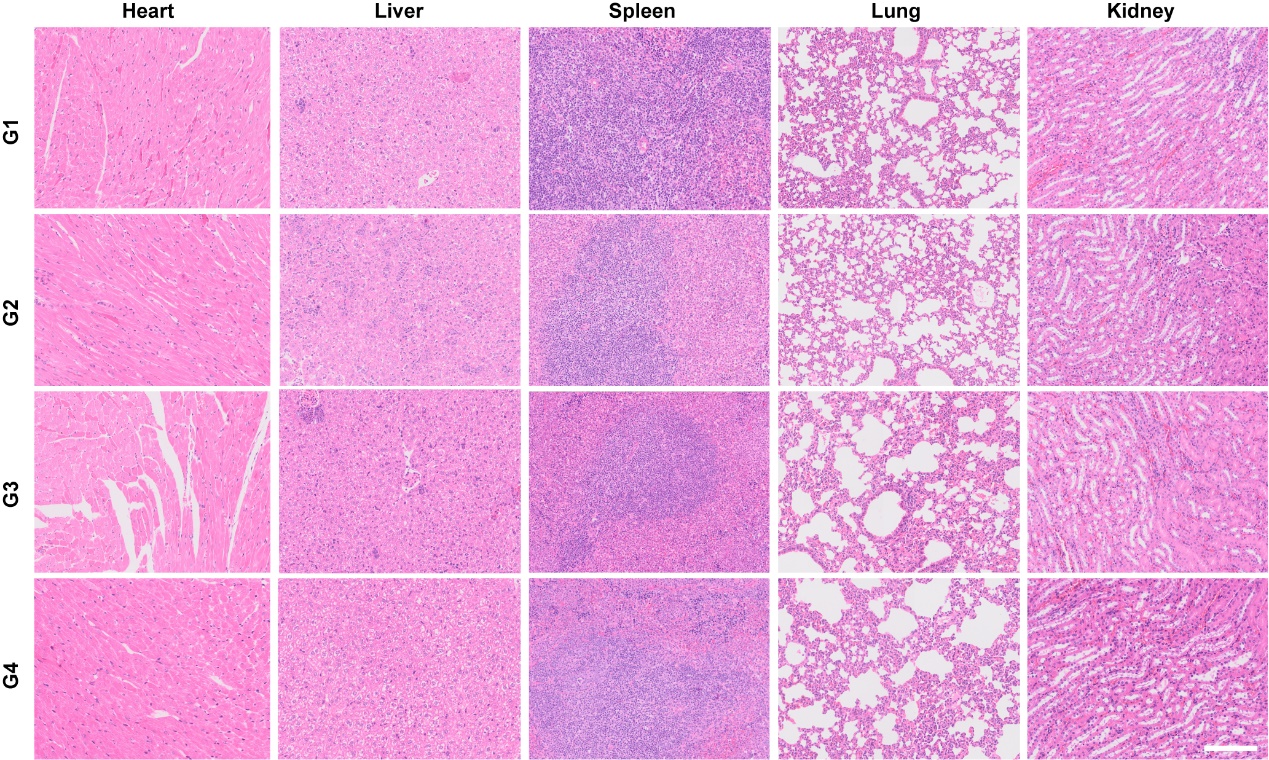


**Figure S26.** Tissues histomorphology was determined by H&E staining to evaluate the therapeutic efficacy and biosafety. (Scale bar=100 μm). G1: Blank MNs, G2: IP10-Az@MNs, G3: DBCO-αCD3/28@MNs, G4: IP10-Az@MNs+DBCO-αCD3/28@MNs.


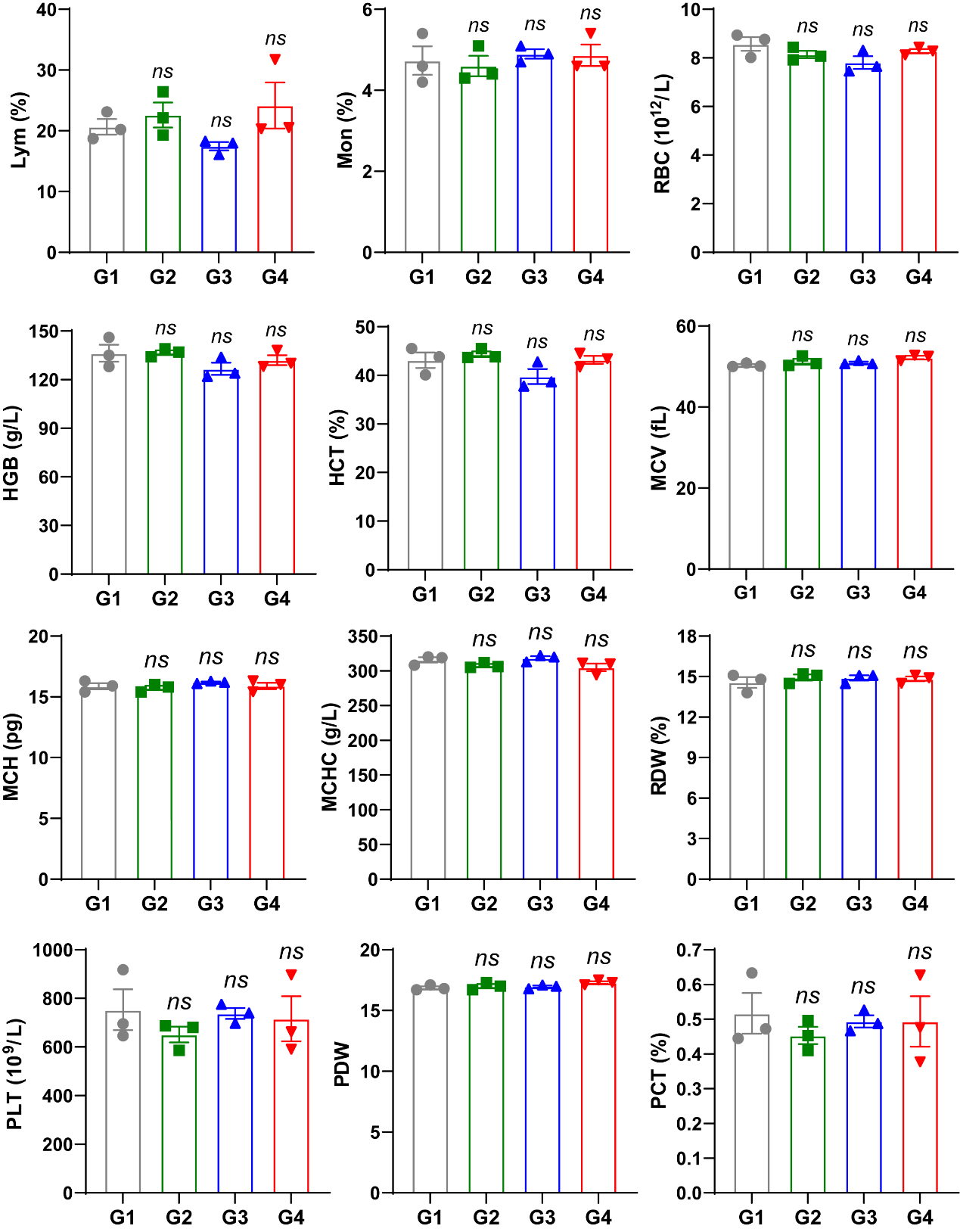


**Figure S27.** Blood routine analysis of 4T1 tumor-bearing mice treated with DBCO-αCD3/28@MNs and IP10-Az@MNs (n=3). Lymph: lymphocytes; WBC: white blood cells, Mon: monocytes; RBC: red blood cells; HGB: hemoglobin; HCT: hematocrit; MCV: mean corpuscular volume; MCH: mean corpuscular hemoglobin; MCHC: mean corpuscular hemoglobin concentration; RDW: red blood cell distribution width; PLT: platelets; PDW: platelet distribution width; PCT: plateletcrit. G1: Blank MNs, G2: IP10-Az@MNs, G3: DBCO-αCD3/28@MNs, G4: IP10-Az@MNs+DBCO-αCD3/28@MNs. *ns*, no significance.


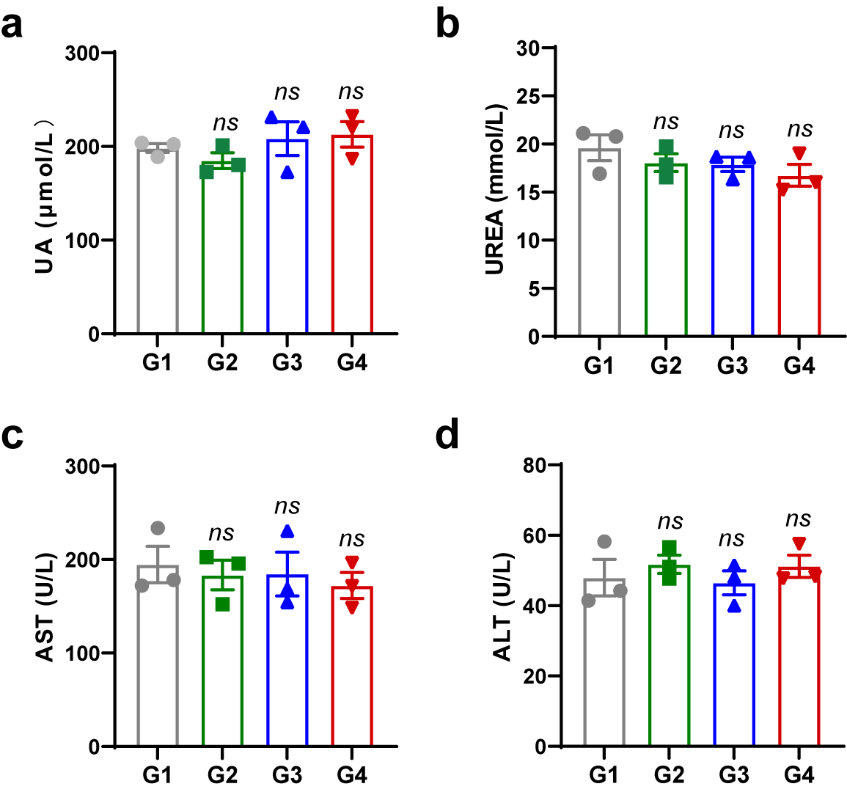


**Figure S28.** Blood biochemistry analysis of 4T1 tumor-bearing mice treated with DBCO-αCD3/28@MNs and IP10-Az@MNs (n=3). UA: uric acid; UREA: creatinine; AST: aspartate aminotransferase; ALT: alanine aminotransferase. G1: Blank MNs, G2: IP10-Az@MNs, G3: DBCO-αCD3/28@MNs, G4: IP10-Az@MNs+DBCO-αCD3/28@MNs. *ns*, no significance.


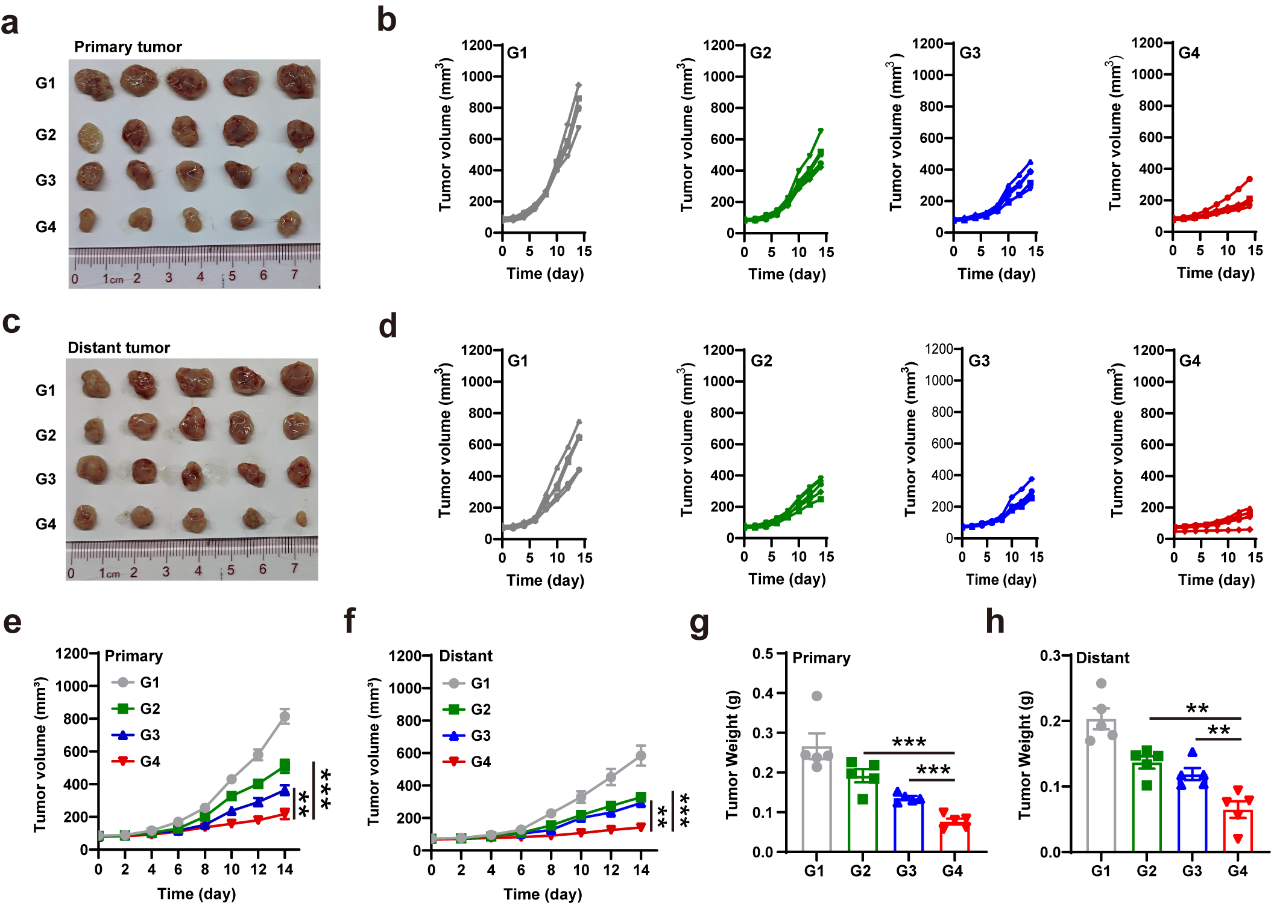


**Figure S29.** a, c) The picture of primary a) and distant c) tumors of 4T1 tumor-bearing mice. b, d) The individual tumor growth curves of primary b) and distant d) tumors. e, f) Average tumor growth curves of primary e) and distant f) tumors. g, h) Average tumor weight of mice primary g) and distant h) tumors (n=5). G1: Blank MNs, G2: IP10-Az@MNs, G3: DBCO-αCD3/28@MNs, G4: IP10-Az@MNs+DBCO-αCD3/28@MNs. ***p* < 0.01, ****p* < 0.001.


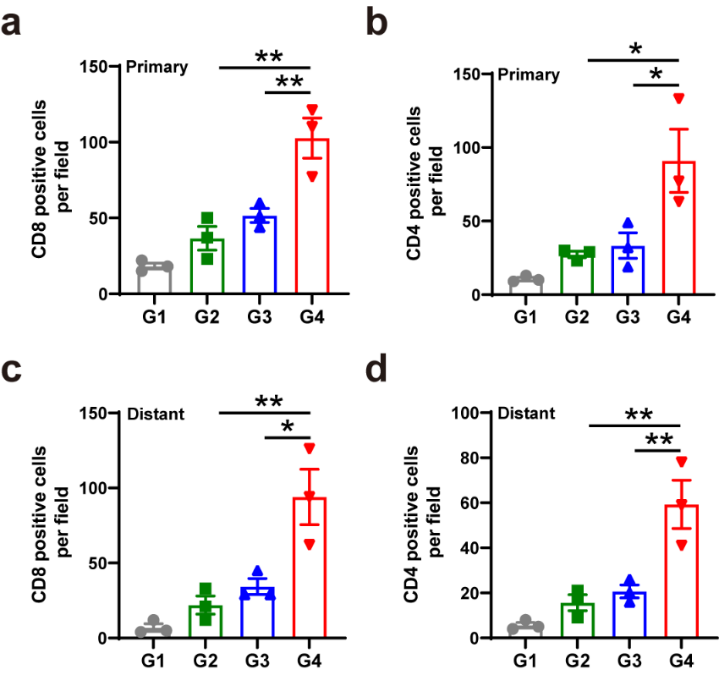


**Figure S30.** Average number of CD8 and CD4 cells per field in primary and distant tumors of 4T1 tumor-bearing mice (n=3). G1: Blank MNs, G2: IP10-Az@MNs, G3: DBCO-αCD3/28@MNs, G4: IP10-Az@MNs+DBCO-αCD3/28@MNs. **p* < 0.05, ***p* < 0.01.


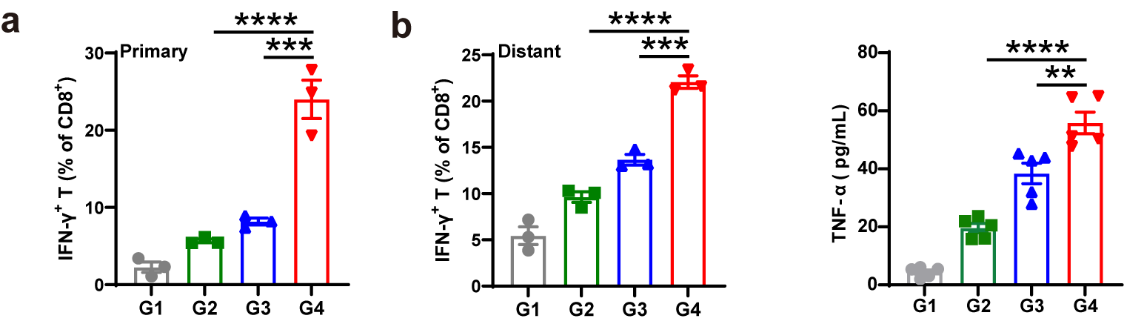


**Figure S31.** Quantification of IFN-γ^+^ CD8^+^ T cells in primary (a) and distant (b) tumors analyzed by flow cytometry (n=3). G1: Blank MNs, G2: IP10-Az@MNs, G3: DBCO-αCD3/28@MNs, G4: IP10-Az@MNs+DBCO-αCD3/28@MNs. ****p* < 0.001, *****p* < 0.0001.


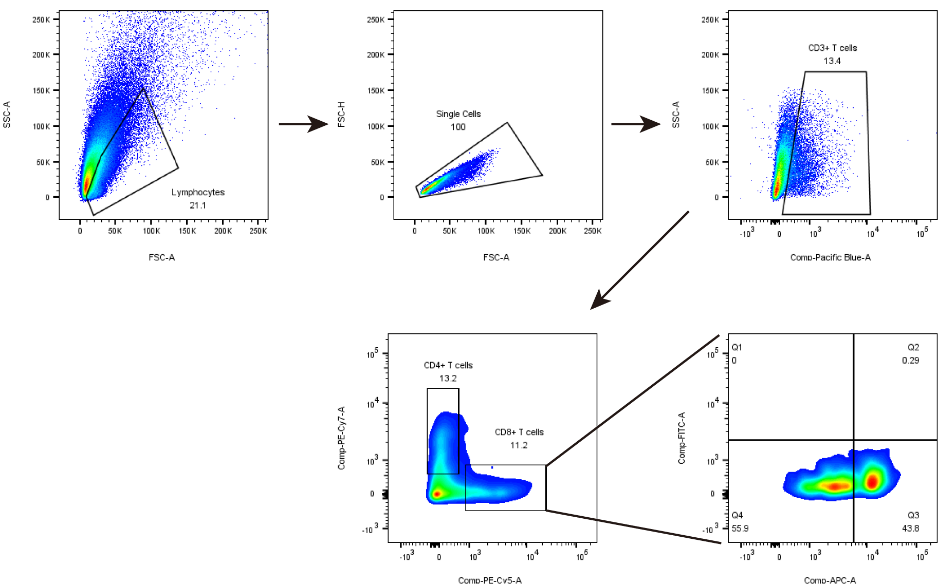


**Figure S32.** A loop-gate flow diagram was constructed to analyze and characterize the cluster of CD44^+^ CD62L^-^ CD8^+^ T cells (gated on CD3^+^/CD4^−^) in tumors.


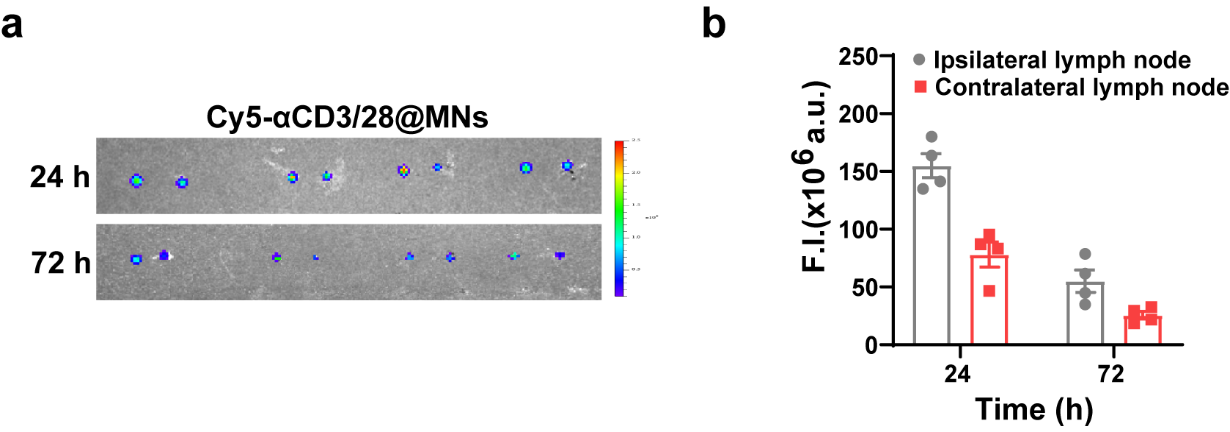


**Figure S33.** a) *Ex vivo* fluorescence imaging of Cy5-αCD3/28 in inguinal lymph nodes on both the ipsilateral and contralateral sides of Cy5-αCD3/28@MNs insertion (one side only) in mice. Cy5-αCD3/28@MNs: MNs loaded with Cy5 labeled αCD3/28. b) Quantification of fluorescence intensity.


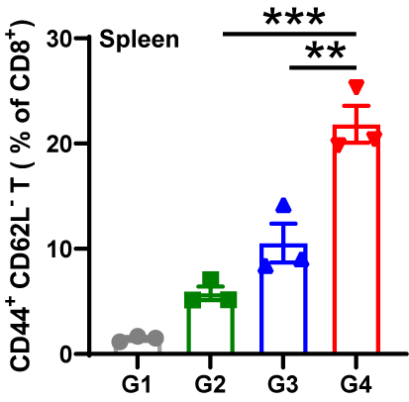


**Figure S34.** Quantification of CD44^+^CD62L^-^ T cells in the spleen of mice analyzed by flow cytometry (n=3). G1: Blank MNs, G2: IP10-Az@MNs, G3: DBCO-αCD3/28@MNs, G4: IP10-Az@MNs+DBCO-αCD3/28@MNs. ***p* < 0.01, ****p* < 0.001.


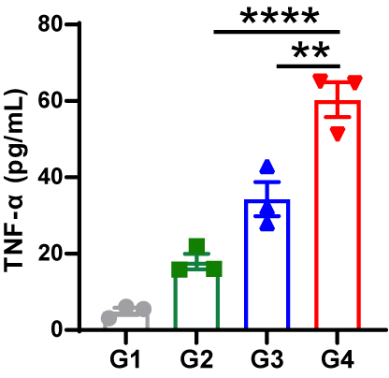


**Figure S35.** Plasma concentration of TNF-α in bilateral tumor-bearing mouse model at the end of treatment (n=3). G1: Blank MNs, G2: IP10-Az@MNs, G3: DBCO-αCD3/28@MNs, G4: IP10-Az@MNs+DBCO-αCD3/28@MNs. ***p* < 0.01, *****p* < 0.0001.


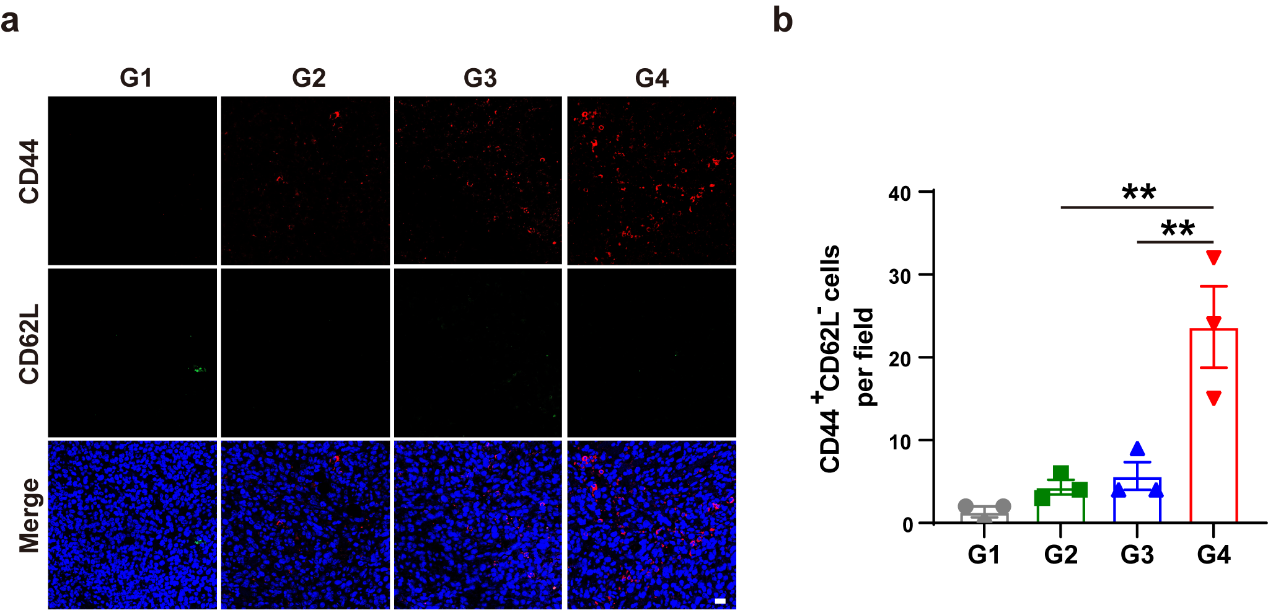


**Figure S36.** a) Representative images of CD44^+^CD62L^-^ cells staining in tumor tissues after different treatments. Scale bar=20 μm. b) Average number of CD44^+^ CD62L^-^ cells per field. G1: Blank MNs, G2: IP10-Az@MNs, G3: DBCO-αCD3/28@MNs, G4: IP10-Az@MNs+DBCO-αCD3/28@MNs. ***p* < 0.01.
